# Nictaba Homologs from *Arabidopsis thaliana* Are Involved in Plant Stress Responses

**DOI:** 10.3389/fpls.2017.02218

**Published:** 2018-01-10

**Authors:** Lore Eggermont, Karolina Stefanowicz, Els J. M. Van Damme

**Affiliations:** Laboratory of Biochemistry and Glycobiology, Department of Molecular Biotechnology, Faculty of Bioscience Engineering, Ghent University, Ghent, Belgium

**Keywords:** plant lectin, Nictaba homolog, *Arabidopsis thaliana*, ArathNictaba, abiotic stress, biotic stress, interaction partner, plant defense

## Abstract

Plants are constantly exposed to a wide range of environmental stresses, but evolved complicated adaptive and defense mechanisms which allow them to survive in unfavorable conditions. These mechanisms protect and defend plants by using different immune receptors located either at the cell surface or in the cytoplasmic compartment. Lectins or carbohydrate-binding proteins are widespread in the plant kingdom and constitute an important part of these immune receptors. In the past years, lectin research has focused on the stress-inducible lectins. The *Nicotiana tabacum* agglutinin, abbreviated as Nictaba, served as a model for one family of stress-related lectins. Here we focus on three non-chimeric Nictaba homologs from *Arabidopsis thaliana*, referred to as AN3, AN4, and AN5. Confocal microscopy of ArathNictaba enhanced green fluorescent protein (EGFP) fusion constructs transiently expressed in *N. benthamiana* or stably expressed in *A. thaliana* yielded fluorescence for AN4 and AN5 in the nucleus and the cytoplasm of the plant cell, while fluorescence for AN3 was only detected in the cytoplasm. RT-qPCR analysis revealed low expression for all three *ArathNictabas* in different tissues throughout plant development. Stress application altered the expression levels, but all three *ArathNictabas* showed a different expression pattern. *Pseudomonas syringae* infection experiments with *AN4* and *AN5* overexpression lines demonstrated a significantly higher tolerance of several transgenic lines to *P. syringae* compared to wild type plants. Finally, AN4 was shown to interact with two enzymes involved in plant defense, namely TGG1 and BGLU23. Taken together, our data suggest that the ArathNictabas represent stress-regulated proteins with a possible role in plant stress responses. On the long term this research can contribute to the development of more stress-resistant plants.

## Introduction

Plants are constantly exposed to multiple abiotic and biotic stresses. The innate immune system of plants encompasses different immune receptors located either at the cell surface or in the cytoplasmic compartment and allows plants to counteract pathogen attack and survive unfavorable conditions. Evidence has been presented that plant lectins play an important role in the plant innate immune system as immune receptors and/or defense proteins (Peumans and Van Damme, 1995; Lannoo and Van Damme, 2010, 2014). Lectins or carbohydrate-binding proteins are widespread in the plant kingdom and can be classified in 12 lectin families (Van Damme et al., 2008). Representative proteins for six out of these lectin families are known as stress-inducible, nucleocytoplasmic lectins. These proteins are low abundant or may even be absent under normal growth conditions, but their expression is elevated when the plant is exposed to stress (Lannoo and Van Damme, 2010).

The Nictaba family represents a family of nucleocytoplasmic lectins and is known to be widespread in the plant kingdom (Delporte et al., 2015; Van Holle et al., 2017). The family was named after the *Nicotiana tabacum* agglutinin (abbreviated as Nictaba), the first lectin of this family (Chen et al., 2002). Nictaba consists of two identical non-covalently linked subunits of 19 kDa (Chen et al., 2002). Hapten inhibition assays revealed the specific interaction of Nictaba with GlcNAc oligomers. In addition, glycan array analyses showed interaction of Nictaba with the core GlcNAc_2_Man_3_ of high-mannose and complex *N*-glycans (Lannoo et al., 2006). Based on sequence alignments and molecular modeling studies Schouppe et al. (2010) predicted that Trp15, Trp22, Glu138, and Glu145 are conserved AA residues in the carbohydrate binding site. Mutational analysis revealed that the tryptophan residues play an important role in the carbohydrate binding activity of Nictaba (Schouppe et al., 2010). Transcript levels for Nictaba were not detectable under normal plant growth conditions but increased several fold after plant exposure to stress situations such as jasmonate treatment and insect herbivory (Chen et al., 2002; Vandenborre et al., 2009). Immunocytochemical localization studies first showed that Nictaba locates to the nucleus and the cytoplasm of leaf parenchyma cells (Chen et al., 2002). Lannoo et al. (2006) confirmed the nucleocytoplasmic localization of Nictaba using fusion constructs with EGFP transiently and stably transformed in different plant systems. Furthermore, using tobacco plants stably expressing a Nictaba promoter-β-glucuronidase fusion construct, Delporte et al. (2013) showed promoter activity in the cotyledons, the leaves as well as the roots of young plants, but promoter activity decreased when the plants grow older. Taking into account the carbohydrate dependent interaction of Nictaba with O-GlcNAc modified core histones, it was suggested that Nictaba might fulfill a role in the remodeling of chromatin conformation, and as such, changing of gene expression in response to stress (Schouppe et al., 2011; Delporte et al., 2014).

The genome of *Arabidopsis thaliana* contains 30 sequences with a Nictaba domain. A large number of these Nictaba homologs possess an N-terminal F-box domain, next to the carbohydrate binding domain. In addition, four Nictaba homologs were identified with an N-terminal Toll/Interleukin-1 receptor domain and one sequence contains an N-terminal avirulence induced gene 1-type G domain (Eggermont et al., 2017). This study will focus on the ArathNictabas containing only a Nictaba domain in order to elucidate the biological importance of this Nictaba domain. Based on protein sequence alignments with Nictaba, the length of the N-terminal domains and identified ESTs three ArathNictabas, in particular AN3, AN4, and AN5, were selected. The localization in the cell, the expression in different plant tissues under normal growth or stress conditions and the interaction partners have been investigated to get insight into the biological importance of these proteins in the stress responses of *A. thaliana*.

## Materials and Methods

### Plant Material and Growth Conditions

Wild type (WT) *A. thaliana* seeds, ecotype Col-0, were purchased from Lehle Seeds (Round Rock, TX, United States). *Arabidopsis* seeds were grown in pots containing commercial soil or in artificial soil (Jiffy-7, 44 mm Ø, distributed by InterGrow, Aalter) in a growth chamber at 21°C with a 16/8 h light/dark photoperiod after a 3 days stratification period at 4°C in the dark. The light intensity in the controlled growth chamber was approximately 100 μmol/m^2^.s [Radium Spectralux plus white (58W) lamps]. Alternatively, seeds were grown *in vitro*, therefore seeds were surface sterilized in 70% ethanol for 2 min followed by 5% bleach for 10 min. Afterward seeds were rinsed with sterile distilled water. The sterilized *Arabidopsis* seeds were sown *in vitro* on solid MS medium (4.3 g/L MS salts with vitamins and nutrients (Duchefa), 30 g/L sucrose [Applichem), pH 5.7-5.8 (adjusted with 0.5 M NaOH) and 8 g/L plant agar (Duchefa)]. After a 3 days stratification period at 4°C in the dark, the plates were transferred to a growth chamber at 21°C with a 16/8 h light/dark photoperiod.

Wild type *N. benthamiana* seeds were supplied by Dr. Verne A. Sisson (Oxford Tobacco Research Station, Oxford, NC, United States). For transient transformation, the tobacco seeds were sown in pots containing commercial soil and cultivated in a growth chamber at 25°C with a 16/8 h light/dark photoperiod.

### Cloning of the *ArathNictaba* Sequences

Plant samples were homogenized using a mortar and a pestle, and total RNA was extracted using TRI Reagent^®^ according to the instructions of the manufacturer (Sigma–Aldrich). The RNA samples were treated with DNase I (Life Technologies) according to the manufacturer’s instructions. The RNA concentration was measured with a Nanodrop 2000 spectrophotometer (Thermo Scientific). cDNA was synthesized from 1 μg of total RNA using the moloney murine leukemia virus RT and oligo(dT)_25_ primer (Life Technologies).

The full length cDNA sequences encoding *AN3*, *AN4*, and *AN5* were retrieved by RT-PCR reactions with gene specific primers (Supplementary Table 1). The PCR reaction mixture was as follows: 2 μl cDNA, 2 μl 10 mM dNTPs (Thermo Fisher Scientific), 2.5 μl 10 × RxN buffer (VWR), 1 μl 5 μM forward and 1 μl 5 μM reverse primer (Life Technologies), 0.75 μl 50 mM MgCl_2_, 0.125 μl Platinum^®^ *Pfx* DNA Polymerase (Life Technologies) and water up to the volume of 25 μl. The PCR conditions used were: 2 min 95°C – 30–35 × (15 s 94°C – 30 s 47–50°C – 1 min 72°C) – 5 s 72°C. After cloning these sequences in the pJET2.1 vector with the CloneJET PCR Cloning kit (Life Technologies), the constructs were checked by agarose gel electrophoresis and sequenced (LGC Genomics, Berlin, Germany) to confirm the correct cDNA sequence.

cDNA quality was checked by RT-PCR using primers specific for the *PP2A* gene (Supplementary Table 2). The PCR reaction mixture was the same as previously mentioned except for the buffer (10 × EXTRA buffer, VWR) and the enzyme (*Taq* DNA polymerase, VWR). The PCR conditions were as follows: 5 min 95°C – 45 × (45 s 94°C – 45 s 55°C – 30 s 72°C) – 5 min 72°C. PCR amplification products were checked by agarose gel electrophoresis.

### Construction of EGFP Fusion Constructs

Coding sequences for the ArathNictabas were N- and C-terminally fused to EGFP using the Gateway^®^ Cloning Technology (Life Technologies, Carlsbad, CA, United States). The cloned full length cDNA sequences (in the pJET1.2 vector) were used as a template to amplify the open reading frames with primers to attach *att*B sites. In the first PCR, the first part of the *att*B site is attached using Platinum^®^ *Pfx* DNA Polymerase (Life Technologies) and primers with a gene specific part (with or without stop codon) and the first part of the *att*B site (Supplementary Table 3). In the second PCR, a 1:5 dilution of the first PCR product was used as a template in combination with primers to complete the *att*B sites (Supplementary Table 3). The PCR conditions for the first PCR were as follows: 2 min 94°C – 30 × (15 s 94°C – 30 s 50°C – 1 min 72°C) – 5 min 72°C. The conditions for the second PCR were: 2 min 94°C – 5 × (15 s 94°C – 30 s 48°C – 1 min 72°C) – 25 × (15 s 94°C – 30 s 55°C – 1 min 72°C) – 5 min 72°C. After checking the PCR products by agarose gel electrophoresis, the PCR fragments were used in a BP recombination reaction with the pDONR221 vector. The *att*B PCR products and the pDONR221 vector were incubated overnight in equimolar amounts with the BP Clonase^®^ II enzyme mix. The next day, the resulting entry clones were transformed into heat-shock competent *Escherichia coli* cells (TOP10) and transformants were selected on LB agar plates with 50 μg/mL kanamycin. Subsequently, transformants were checked with colony PCR and agarose gel electrophoresis. The entry clones were extracted using the GeneJET Plasmid Miniprep kit (Thermo Fisher Scientific) according to the manufacturer’s instructions. After sequencing (LGC Genomics, Berlin, Germany) the entry clones were used in a LR recombination reaction with the destination vectors pK7WGF2,0 or pK7FWG2,0 to make the N-terminal and C-terminal fusion constructs with EGFP, respectively (Karimi et al., 2002). This recombination reaction was incubated overnight according to the Gateway^®^ manual and expression clones were transformed into *E. coli* TOP10 cells using heat shock. Transformants were selected on LB agar plates with 75 μg/mL spectinomycin and screened with colony PCR using gene specific and EGFP primers (Supplementary Table 3).

The expression vectors containing the different EGFP fusion constructs were introduced into *Agrobacterium tumefaciens* C58C1 pMP90 cells using triparental mating. Briefly, a donor strain (*E. coli* containing the expression vectors), a helper strain and the *Agrobacterium* were mixed together on solid YEB medium (5 g/L beef extract, 5 g/L peptone, 1 g/L yeast extract, 5 g/L sucrose, and 15 g/L bacterial agar) containing 2 mM MgSO_4_. After incubation, dilution series were made and transformants were selected on YEB medium containing 75 μg/mL spectinomycin and 20 μg/mL gentamicin. After purification of the expression clones from the *Agrobacterium*, screening of the clones was done by PCR (Supplementary Table 3).

### Construction of *ArathNictaba* Overexpression Constructs

The entry clones containing the *AN4* and *AN5* coding sequences with stop codon (see section “Construction of EGFP Fusion Constructs”) were used in an LR reaction to generate the overexpression constructs. The destination vector pK7WG2,0 (Karimi et al., 2002) containing the 35S promoter was combined with the entry clones and the Gateway LR Clonase II to get the desired expression clones (35S::*AN4* and 35S::*AN5*). These expression clones were heat shock transformed in TOP10 *E. coli* cells and transformants were selected on LB agar plates containing 75 μg/mL spectinomycin. Transformants were screened with colony PCR using a forward primer in the 35S promoter and a reverse primer in the 35S terminator sequence (Supplementary Table 4). Electrocompetent *A. tumefaciens* GV3101 cells were transformed with these expression clones (300 ng) using electroporation with the following parameters: 2.0 kV, 25 μF, and 200 Ω. Immediately after the pulse, YEB medium was added and the cells were grown on a shaker (200 rpm) at 28°C for 2 h. Transformants were selected on YEB agar plates with 75 μg/mL spectinomycin and screened with colony PCR using primers located in the 35S promoter and terminator sequences (Supplementary Table 4).

### Transient Transformation of *N. benthamiana* Leaves

Transient expression of the EGFP fusion constructs was obtained by infiltration of the transformed *Agrobacterium* in leaves of 4- to 6-week-old *N. benthamiana* plants as described by Sparkes et al. (2006). First, the *Agrobacterium* strains were grown in liquid YEB medium containing 75 μg/mL spectinomycin and 20 μg/mL gentamicin for 2 days at 25°C on a rotary shaker (200 rpm). *Agrobacterium* cells were harvested by centrifugation and resuspended in infiltration medium (50 mM MES, 2 mM Na_2_HPO_4_, 0.5% glucose, pH 5.6). Centrifugation and resuspension were repeated twice, the second time using infiltration medium with 100 μM acetosyringone. After washing, the cells were diluted to a final optical density at 600 nm of 0.01, 0.05, 0.1, and 0.2 and infiltrated in the leaf epidermal cells. Two or three days post-infiltration, microscopic analysis was performed.

### Stable Transformation of *A. thaliana* Plants

Stably transformed *Arabidopsis* plants were created using the floral dip transformation method (Clough and Bent, 1998). Transformed seeds were selected on MS medium containing 75 μg/mL kanamycin (Duchefa) using the fast selection protocol according to Harrison et al. (2006). Green plantlets were transferred to new selective MS medium and afterward to artificial soil. For the ArathNictaba EGFP overexpression lines integration of the T-DNA was checked by PCR on gDNA using gene specific and EGFP primers (Supplementary Table 3). T2 generation *Arabidopsis* plants were used for all analyses. For the ArathNictaba overexpression lines, selection was done until the transformed plants were homozygous for the T-DNA integration (T3 and T4). Integration of the T-DNA was checked by PCR on gDNA using kanamycin resistance gene primers (Supplementary Table 4) using the following PCR program: 10′ 94°C – 45 × (30′′ 94°C – 30′′ 48°C – 1′ 72°C) – 5′ 72°C. The quality of the gDNA was checked with *actin* primers (Supplementary Table 4) using the same PCR program. Overexpression levels of the *ArathNictaba* genes in 15-day-old seedlings were quantified by RT-quantitative (q)PCR. Three independent homozygous single insertion lines were selected for each construct (35S::*AN4* and 35S::*AN5*).

### ArathNictaba Expression during Plant Development

For the aerial plant tissues, WT *Arabidopsis* seeds were sown *in vitro* on MS medium (until 22 days) or in artificial soil (Jiffy-7, 44 mm Ø). The *Arabidopsis* plants grown in artificial soil were watered regularly and fertilizer was added once after 25 days. Whole plantlets were collected at 6, 15, and 22 days after sowing. Rosette leaves from at least five plants were harvested and pooled after 31 days. After 39 and 54 days, rosette leaves, cauline leaves, stems and flowers from at least five plants were sampled and pooled. For the root samples, WT *Arabidopsis* seeds were sown in expanded clay granules (Ø < 4 mm). The plants were watered regularly and fertilizer was added once per week. Root samples from at least 20 plants were collected and pooled after 34, 46, and 59 days. All samples were immediately frozen in liquid nitrogen and stored at -80°C prior to RNA extraction. Two biological replicates were performed and analyzed, each with two technical replicates.

### Hormone and Abiotic Stress Treatments

Sixteen-day-old *Arabidopsis* seedlings grown *in vitro* on a filter paper on top of MS medium were used for the treatment with the following solutions: 100 μM MeJA, 100 μM ABA, 300 μM SA, and 150 mM NaCl. Prior to use, stock solutions of the hormones (MeJA, ABA, and SA) were made in 100% ethanol and water was used in case of the salt solution. Control plants were kept on liquid MS medium containing an equal concentration of the corresponding solvent (ethanol or water). For each treatment, the filter papers with the germinated seedlings were transferred to Petri dishes filled with liquid MS medium containing either the hormone or the salt solution, and incubated at 21°C. Heat stress was applied by incubating the plates with seedlings in the dark at 37°C, controls were incubated at 21°C in the dark. For every treatment, 50 seedlings were collected at several time points (1, 3, 5, 10, and 24 h) after stress initiation. Samples were immediately frozen in liquid nitrogen and stored at -80°C prior to use. Four independent biological replicates were performed for MeJA, SA and NaCl stress, two biological replicates were performed for ABA and heat stress.

### Biotic Stress Treatments

*Pseudomonas syringae* and *Botrytis cinerea* infection experiments were performed with 5-week-old WT *Arabidopsis* plants of the Col-0 ecotype grown in artificial soil (Jiffy-7). The *P. syringae* pv. *tomato* DC3000 strain and the *B. cinerea* B05.10 strain were supplied by Prof. Dr. M. Höfte of the Phytopathology Lab (Ghent University, Belgium). Infection assays were performed according to Pieterse et al. (1996), Audenaert et al. (2002), and Katagiri et al. (2002), with minor modifications.

*Pseudomonas syringae* pv. *tomato* DC3000 was grown in liquid King’s B medium (20 g/L peptone, 1% glycerol, 1.5 g/L KH_2_PO_4_, 1.5 g/L MgSO_4_⋅7H_2_O, pH 7.2) at 28°C on a rotary shaker (200 rpm) until the culture reached the mid to late log growth phase (OD_600_ = 0.6–1.0). After centrifugation of the culture (10 min, 2500 g), bacterial cells were resuspended in 10 mM MgSO_4_ to obtain a solution of bacteria with an OD_600_ of 0.05 (corresponding to 2.5 × 10^7^ cfu/mL). Prior to use, 0.05% Silwet-77 (GE Specialty Materials, Switzerland) was added to the infection solution. The mock solution consisted of 10 mM MgSO_4_ containing 0.05% Silwet-77. The rosette leaves of the *Arabidopsis* plants were sprayed until run-off with either the infection or the mock solution. To increase the efficiency of the infection, the plants were maintained at 100% relative humidity 1 day before the treatment until 2 days after the start of the bacterial infection. Three biological replicates were performed and analyzed.

The *Botrytis* strain was kept on regular potato dextrose agar plates at 21°C. Sporulation was stimulated by incubation for 10 days at 21°C under a 12/12 h UV/dark light regime. After 10 days, *Botrytis* spores were harvested by washing the plates with distilled water containing 0.01% Tween-20 (VWR). This suspension was filtered through a nylon membrane (20 μm Ø) and an inoculation suspension (5 × 10^5^ conidia/mL) was prepared in half strength potato dextrose broth medium. The mock solution consisted of the same medium without spores. Droplets (10 μL) of the infection suspension or the mock solution were applied on the upper side of three rosette leaves from each plant. Plants were maintained in 100% relative humidity during the entire experiment. Two biological replicates were performed and analyzed.

During the infection assays, plants were kept in a Conviron growth chamber at 21°C with a 12/12 h light/dark photoperiod. Control plants were kept separately from infected plants. Rosette leaves of 8-10 randomly selected plants were sampled in liquid nitrogen at different time points post-infection and samples were stored at -80°C prior to use.

*Myzus persicae* was kindly provided by Prof. Dr. Guy Smagghe (Agrozoology Lab, Ghent University, Belgium) and kept on sweet pepper plants under lab conditions (Shahidi-Noghabi et al., 2009). Aphid infestation was performed on 5-week-old *Arabidopsis* plants sown in round plastic pots (Ø 11 cm) with soil on which a transparent ventilated cage (Novolab) was placed. Sixty aphids were placed on the rosette leaves of each plant. Control plants were grown in cages without aphids. During the assay, plants were kept in a Conviron growth chamber at 21°C with a 12/12 h light/dark photoperiod. At indicated time points, two leaves of nine randomly chosen plants were sampled, frozen in liquid nitrogen and stored at -80°C prior to use. Four biological replicates were performed and analyzed, each with two technical replicates.

### RT-qPCR Analysis

Real time RT-qPCR analyses of the gene expression during development and after *P. syringae* infection, were performed using the Rotor-Gene 3000 (Corbet Life Science) and the Rotor Discs (Qiagen, Hilden, Germany). The program was as follows: 10 min 95°C – 45 × (25 s 95°C – 25 s 60°C – 20 s 72°C) – 5 min 72°C followed by generation of a melting curve (gradual increase from 72 to 95°C with 1°C/step). The Rotor Gene 6 software generated the raw output data (*C*q values), these were statistically analyzed using the REST-384 software (Corbett Research). REST-384 uses a pair wise fixed reallocation randomization test as a statistical model (Pfaffl et al., 2002).

RT-qPCR analyses of the other stress experiments and overexpression experiments were performed using the 96-well CFX Connect^TM^ Real-Time PCR Detection System (Bio-Rad). The program was as follows: 10 min 95°C – 45 × (15 s 95°C – 25 s 60°C – 20 s 72°C) followed by generation of a melting curve (gradual increase from 65 to 95°C with 0.5°C/step). The CFX Manager 3.1 software (Bio-Rad) generated the raw output data which were statistically analyzed using the REST-384 software.

All reactions were conducted in a total volume of 20 μl containing 1 × SensiMix^TM^ SYBR^®^ No-ROX One-Step mix, 2 μl undiluted cDNA template, 500 nM gene specific forward and reverse primers (Supplementary Table 2). All gene specific qPCR primers were designed using Primer3^1^. Specificity of the primers was tested *in silico* by BLAST search and amplicons were cloned and verified using agarose gel electrophoresis and sequencing (LGC Genomics, Berlin). Amplification efficiency of all primer pairs was determined in the CFX Manager 3.1 (Bio-Rad) and qBASE^PLUS^ software (Hellemans et al., 2007). All expression data were normalized using three reference genes: *PP2A*, *TIP41*, and *UBC9* (Czechowski et al., 2005). All melting curves were checked and reference gene stability and quality control of the samples were validated in the qBASE^PLUS^ software (Hellemans et al., 2007).

### *P. syringae* Infection of *Arabidopsis* Overexpression Lines

Four-week-old *Arabidopsis* WT plants and three independent homozygous transgenic lines for each construct (35S::*AN4* and 35S::*AN5*) were inoculated with the infection (1.6 × 10^7^ cfu/mL) or mock solutions. Rosette leaves of three individual randomly selected plants were sampled at different time points post-infection (1 – 5 dpi). Two independent biological replicates were performed.

To measure leaf damage, six leaves per line per time point were scanned with a flatbed scanner at the highest resolution. The percentage of leaf damage was determined using the Image Analysis Software for Plant Disease Quantification Assess 2.0 (APS, St. Paul, MN, United States) using a self-written macro adjusted to our sampled leaves. The data were tested for normal distribution with the Shapiro–Wilkinson test. The Mann–Whitney *U* test was used for not-normally distributed data, supplemented with a non-parametric equivalent of the Levene’s test to check homogeneity of variances. The Bonferroni–Holm correction was used for multiple testing.

Using trypan blue staining (Sigma–Aldrich, Diegem, Belgium), plant cell death was visualized. One leaf of three plants per line for each time point was submerged in trypan blue solution (0.02%) and boiled for 2 min. Afterward, the leaves were incubated overnight at room temperature on a rotary shaker (50–100 rpm). Next, the trypan blue solution was replaced with a chloral hydrate solution (100 g/40 mL water) to destain the leaves. The destained leaves were placed on a microscopy slide in 50% glycerol and pictures were taken with a Leica S8APO microscope (DFC400 camera) and Leica Plan APO 1.6× objective. The trypan blue staining was scored by estimating the percentage of blue staining or cell death. Leaves without trypan blue staining (0%) were assigned a score 1. Score 2 was assigned to leaves for which the percentage of cell death was 1–30%. Leaves with 31–60% of cell death were assigned a score 3. Score 4 was assigned to leaves for which the percentage of cell death was 61–100%. Each transgenic line was statistically compared with the WT using a Mann-Whitney *U* test supplemented with a non-parametric equivalent of the Levene’s test to check homogeneity of variances. The Bonferroni–Holm correction was used for multiple testing.

To determine the *P. syringae* biomass, gDNA was first extracted from the infected and mock treated leaves using CTAB. The CTAB buffer (2% CTAB, 0.1 M Tris-HCl pH 7.5, 1.4 M NaCl, 20 mM EDTA) was added to 100 mg of plant material and this mixture was incubated at 65°C for 90 min in a shaking heat block. After chloroform:isoamylalcohol (24:1) extraction the gDNA was precipitated with 100% isopropanol, washed with 76% ethanol/0.2 M NaOAc and 76% ethanol/10 mM NH_4_OAc and dissolved in water. Quantification of *P. syringae* biomass was performed with RT-qPCR using *oprf* primers targeting the outer membrane porin protein F gene of *P. syringae* (Brouwer et al., 2003) (Supplementary Table 2). *ACT2* and *PEX4* primers were used as reference genes for *A. thaliana* (Supplementary Table 2). The REST-384 software was used to calculate the ratio of *P. syringae* gDNA to *A. thaliana* gDNA (Pfaffl et al., 2002).

### Recombinant Protein Expression AN4

Gibson assembly was used to assemble the AN4-HIS sequence with the pET-21a(+) vector (Novagen) (Gibson et al., 2009). The AN4 open reading frame followed by a Gly3-linker and a His6-tag was used as a template for PCR. Forward and reverse primers were used to add Gibson assembly sites (Supplementary Table 5). The vector backbone of the pET21a vector was amplified in a PCR reaction with the following components: 5 × Q5 reaction buffer, 2 mM dNTP mix, 0.1–1 ng pET21a, Q5 high fidelity DNA polymerase, water, forward and reverse primer (10 μM) (Supplementary Table 5). Linearization of the vector backbone was performed by *Dpn*I restriction (1 h at 37°C). PCR products were purified with the InnuPREP PCR pure kit (Analytik Jena, Germany). For the Gibson assembly reaction, equimolar amounts of linear vector backbone and AN4 expression construct were mixed together with 15 μl of Gibson master mix and incubated at 50°C for 1 h.

Half of the Gibson assembly mixture was transformed into heat shock competent *E. coli* strain Rosetta(DE3) cells (Novagen). Transformants were grown on LB agar plates supplemented with 100 μg/mL ampicillin and 25 μg/mL chloramphenicol, and screened by colony PCR using primers that contain the Gibson assembly sites (Supplementary Table 5). The pET21a plasmids containing the AN4-HIS were purified using the GeneJET Plasmid Miniprep kit (Life Technologies) and sequenced by LGC Genomics (Berlin, Germany) with a forward sequencing primer on pET21a (Supplementary Table 5).

Recombinant *E. coli* Rosetta(DE3) cells were grown overnight in 5 mL LB supplemented with 20 μg/mL ampicillin and 25 μg/mL chloramphenicol at 37°C on a rotary shaker (185 rpm). The next morning, the *E. coli* cells were diluted in 50 ml LB supplemented with 200 μg/mL carbenicillin and 25 μg/mL chloramphenicol, and grown at 30°C on a rotary shaker (185 rpm). After the cells reached an OD_600__nm_ of 0.5–0.6, the expression of recombinant protein was induced with 0.2 mM IPTG. After application of IPTG, the *E. coli* cells were grown overnight at 14°C on a rotary shaker (185 rpm). When growing bacterial cultures in larger volumes (300 mL), the time of induction was prolonged to 3 days to obtain more recombinant protein. Next, the *E. coli* cell cultures were harvested by centrifugation at 8,000 rpm for 15 min. The cell pellets were kept overnight at -20°C and solubilized in 10 mL of 1 × PB (20 mM NaH_2_PO_4_⋅2H_2_O, 230 mM Na_2_HPO_4_) with 1 M NaCl and 1 mg/mL lysozyme at pH 8.0. Additionally, the cells were sonicated three times for 2.5 min. After sonication, the solutions were centrifuged at 4°C for 45 min (9,000 rpm) to separate the soluble from the insoluble fractions. The insoluble fractions were resuspended in 8 M urea. Both fractions were checked for the presence of recombinant protein by SDS - PAGE and Western blot analysis.

*Escherichia coli* cell extracts containing the recombinant AN4-HIS were used for further purification. Therefore extracts adjusted to 10 mM IZ and pH 8.0, were loaded on a Ni-NTA agarose column (MCLAB, South San Francisco, CA, United States) equilibrated with PB pH 8.0 containing 1 M NaCl. The column was washed with PB containing 1 M NaCl, 50 mM IZ, pH 8.0 and PB containing 1 M NaCl, 75 mM IZ, pH 8.0 to remove a specifically bound proteins. Elution of the column was performed with PB containing 1 M NaCl, pH 8.0 with increasing concentrations of IZ ranging from 100 to 500 mM IZ. Several fractions of 500 μL were collected for each elution buffer. The OD_280__nm_ of each fraction was measured with the Nanodrop 2000 spectrophotometer (Thermo Scientific). The purity of the protein samples was verified by SDS-PAGE and Western blot analysis.

Protein samples were analyzed by SDS-PAGE on 15% acrylamide gels as described by Laemmli (1970). After separation, proteins were visualized by gel staining with Coomassie Brilliant Blue R-250 or blotted onto polyvinylidene fluoride transfer membranes (FluoroTrans^®^ PVDF, Pall Laboratory, United States). Membranes were blocked with TBS (10 mM Tris, 150 mM NaCl, 0.1% (v/v) Triton X-100, pH 7.6) containing 5% (w/v) non-fat milk powder. Subsequently, membranes were incubated for 1 h with a mouse monoclonal anti-His6 antibody (Thermo Fisher Scientific) diluted 1:3000 in TBS. After washing three times with TBS, membranes were incubated for 1 h with the 1:1000 diluted rabbit anti-mouse IgG secondary antibody labeled with horseradish peroxidase (Dako, Glostrup, Denmark). After washing twice with TBS and once with 0.1 M Tris-HCl buffer (pH 7.6), immunodetection was achieved using a colorimetric assay with 0.1 M Tris-HCl buffer (pH 7.6) containing 700 μM 3,3′-diaminobenzidine tetrahydrochloride (Sigma–Aldrich, St. Louis, MO, United States) and 0.03% (v/v) hydrogen peroxide. The detection reaction was stopped after 2–10 min by washing the membrane with distilled water. All washes and incubations were performed at room temperature on a gently shaking platform.

### Pull-down Analysis

Pull-down assays were performed on column. First, the soluble fractions of the two different *E. coli* cultures harboring the AN4-HIS pET expression vector (treated or non-treated with IPTG) were incubated with the Ni matrix overnight. After washing, the Ni-agarose beads were incubated with leaf extracts from 16-day-old *Arabidopsis* plants subjected or not subjected to NaCl stress for 5 h, as described in Section “Hormone and Abiotic Stress Treatments,” resulting in four different combinations. For each pull-down experiment three replicates were performed and analyzed by mass spectrometry.

First, the soluble fraction (50 mL) of the *E. coli* culture producing recombinant AN4-HIS was incubated overnight with the equilibrated Ni matrix (100 μL). The soluble fraction originated from a 300 mL *E. coli* culture induced or non-induced with IPTG to produce the recombinant AN4 (bait). After overnight incubation, the mixture of Ni-agarose beads and soluble *E. coli* protein solution was transferred in a Poly-Prep purification column (Bio-Rad). The column was washed first with PB containing 1 M NaCl and 50 mM IZ at pH 8.0 followed by a second wash with the same buffer, but with 75 mM IZ. Then the Ni-agarose beads were transferred to an Eppendorf tube, 1 mL plant extract (prey) was added to the beads and this mixture was incubated for 30–35 min on a turning wheel. The plant extract was obtained from untreated or salt-stressed *Arabidopsis* plant material (0.5 mL) with 1 mL extraction buffer (1 × PB, 1 M NaCl, 25 mM IZ, 10% glycerol, 0.1% Tween-20, 1% β-mercaptoethanol and complete^TM^, Mini, EDTA-free Protease Inhibitor Cocktail at pH 8.0). After centrifugation and removal of the plant extract, the beads were washed with the first wash buffer containing 50 mM IZ. Finally, the beads were washed three times with 20 mM Tris-HCl containing 2 mM CaCl_2_ at pH 8.0 and resuspended in 150 μL of the same buffer to store at -20°C prior to mass spectrometry. All steps described above were performed in a cold room at 4°C.

Before mass spectrometry, the samples were treated with trypsin (1 μg) for 4 h at 37°C to cleave all proteins from the Ni beads. After removal of the Ni beads, a second trypsin treatment was performed overnight at 37°C. Trifluoroacetic acid (1%) was added to deactivate the trypsin and the samples were subsequently desalted. Next, the samples were dried completely and re-dissolved in 2% acetonitrile and 0.1% trifluoroacetic acid. Afterward, mass spectrometry was performed with the Q Exactive^TM^ HF Hybrid Quadrupole-Orbitrap^TM^ Mass Spectrometer. Database searches were achieved with the MaxQuant software and statistical analysis with the Perseus software. Both analyses were outsourced to the Proteomics Expertise Center (Center for Medical Biotechnology, VIB, UGhent).

### Confocal Microscopy and Image Analysis

Microscopy analysis was performed using the confocal laser scanning microscope Nikon A1R (Nikon Belux). The lower epidermis of the leaf disks (spots that are infiltrated) were visualized using the 40 × S Plan Fluor ELWD objective lens (NA 0.6). All images are a combination of different fluorescent images acquired along the *z*-axis, as such the complete epidermis cell could be visualized. EGFP was excited with a 488 nm argon ion laser, fluorescent emission filters were 525 nm for EGFP and 650 nm for autofluorescence of chlorophyll. All images were created by the software package NIS-Elements (Nikon) and image analysis was performed using Fiji (Schindelin et al., 2012).

### *In Silico* Tools

Multiple sequence alignments were performed with Clustal Omega^2^ and pairwise sequence alignments were obtained using EMBOSS Water^3^. Prediction of the presence of a signal peptide was conducted using Phobius and SignalP 4.1 (Käll et al., 2004; Petersen et al., 2011), whereas the presence of a NLS was predicted by NucPred (Brameier et al., 2007). Using SUBA3, protein subcellular localization was predicted (Hooper et al., 2014). BLASTn and tBLASTn searches against the expressed sequence tag (EST) database were performed using the NCBI website^4^. Using the eFP browser (Winter et al., 2007) and Genevestigator (Hruz et al., 2008), microarray expression data were analyzed to study the developmental expression as well as the gene expression under different stress conditions.

## Results

### Nictaba Homologs in *A. thaliana*

**FIGURE 1:**
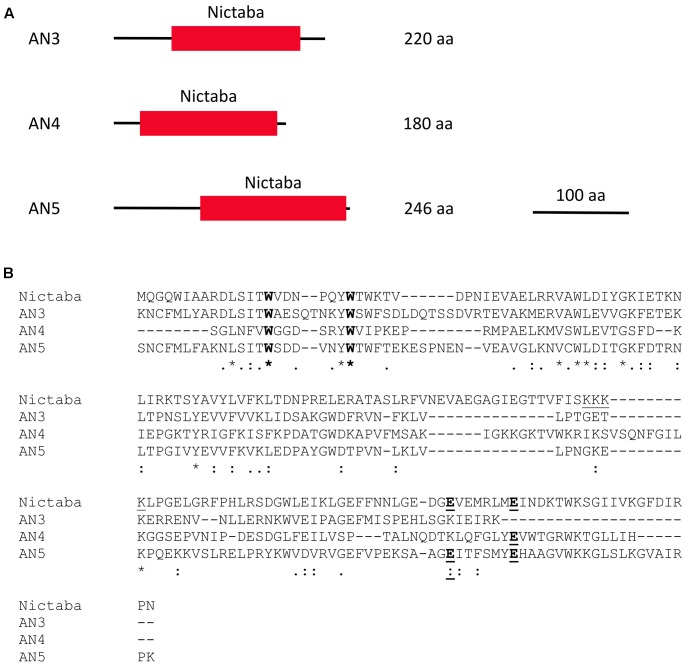
Domain architectures of AN3, AN4, and AN5 **(A)**, and protein sequence alignment of the Nictaba sequence and the Nictaba domains of the ArathNictabas **(B)**. **(A)** The domain architectures are drawn to scale. The scale bar represents 100 AA. **(B)** The tryptophan residues important for carbohydrate binding in Nictaba are marked in bold (Trp15, Trp22). The glutamic acid residues (Glu138 and Glu145) are marked in bold and underlined. The NLS of Nictaba is underlined.

This study focuses on three Nictaba homologs from *Arabidopsis*, namely AN3 (AT4G19850.2), AN4 (AT1G31200), and AN5 (AT4G19840). These ArathNictaba sequences contain next to their Nictaba domain (approximately 145 AA), an N-terminal sequence of different length (**Figure 1A**). Protein BLAST with these N-terminal sequences concedes no homology to any known protein domain. A search for signal peptides and transmembrane regions using the Phobius and SignalP 4.1 server predicted no signal peptides or transmembrane regions. Judging from the absence of signal peptides, AN3, AN4, and AN5 are probably synthesized on free ribosomes in the cytoplasm.

Assuming that the primary transcripts undergo no further processing, AN3, AN4, and AN5 will differ from each other in their molecular masses and isoelectric points. AN5 is the largest protein with a molecular mass of 28.1 kDa. AN3 has a molecular mass of 25.6 kDa and AN4 is the smallest protein, being 20.3 kDa. AN3 is an acidic protein with an isolectric point of 6.11 whereas both AN4 and AN5 have an isoelectric point around 9 (9.44 and 9.18, respectively).

Sequence alignment of the Nictaba domains for the ArathNictaba 3–5 and Nictaba from tobacco (encoded by AF389848) allowed to calculate the sequence identities and similarities (**Figure 1B**). AN3 and AN5 show the highest percentage of sequence identity (48.4% sequence identity; 67.5% sequence similarity) in the lectin domain sequence. Moreover, their sequence identity with Nictaba is very similar, 31.8 and 31.5%, respectively. The lowest percentage of sequence identity with Nictaba is observed for AN4 (22.9%). The tryptophan residues reported to be important for carbohydrate binding activity of Nictaba are conserved in all ArathNictaba sequences (**Figure 1B**, Schouppe et al., 2010).

### ArathNictabas Locate to the (Nucleo)Cytoplasmic Compartment

**FIGURE 2:**
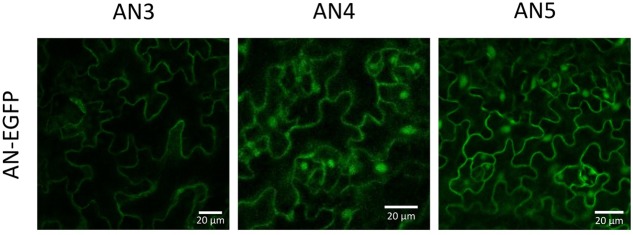
Localization of ArathNictaba-EGFP fusion constructs expressed in stably transformed *Arabidopsis thaliana* plants. Localization is shown in the lower epidermis cells of the leaves. All images are a compilation of different fluorescent images acquired along the *z*-axis.

To validate the *in silico* results with respect to the cellular localization, N- and C-terminal EGFP fusion constructs were created. Microscopy analysis of the T2 generation of *Arabidopsis* plants stably transformed with the C-terminal EGFP fusion constructs for each ArathNictaba sequence showed a nucleocytoplasmic localization for AN4 and AN5 whereas AN3 only resided in the cytoplasm (**Figure 2**). The localization of AN4 and AN5 in the cytoplasm and the nucleus was confirmed for the N-terminal EGFP fusion constructs. The N-terminal EFGP fusion construct of AN3 never showed a fluorescent signal (Supplementary Figure 1A).

In addition, leaves from *N. benthamiana* were transiently transformed with the N- and C-terminal EGFP fusion constructs for each ArathNictaba sequence and confirmed the results obtained with *A. thaliana* (Supplementary Figure 1B). In both experiments, free EGFP was used as a positive control and was observed in the nucleus and the cytoplasm (Supplementary Figures 1A,B).

### Expression of the *ArathNictaba* Genes during Development of WT *A. thaliana* Plants

**FIGURE 3:**
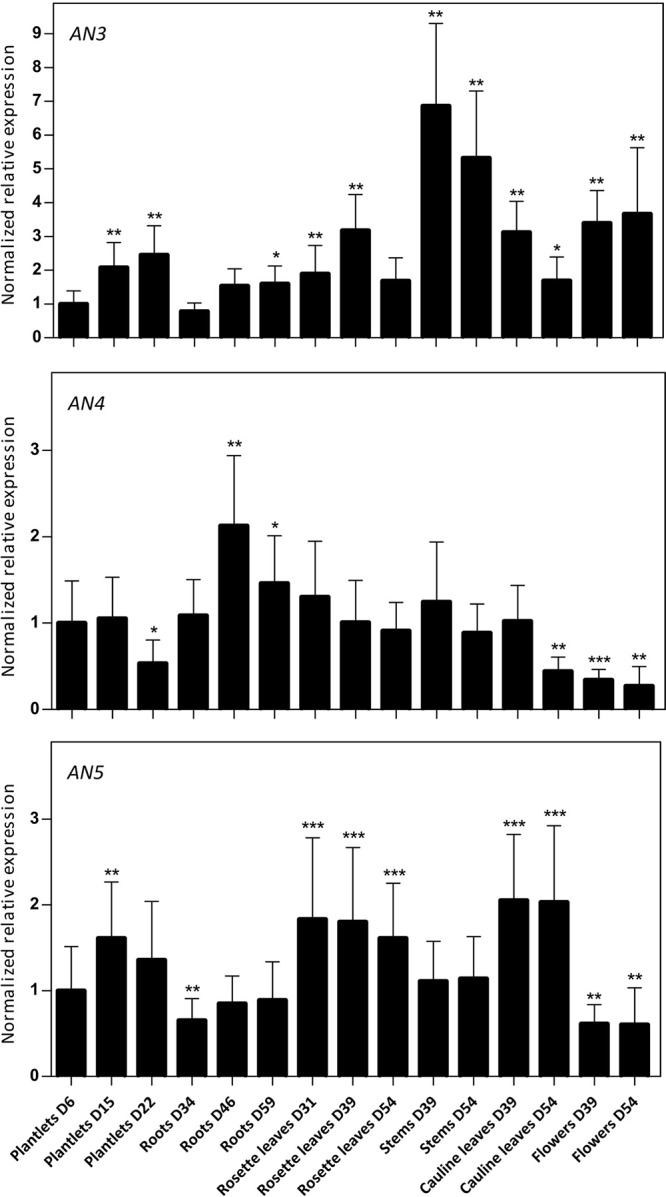
Normalized relative expression of *ArathNictaba* genes during the development of *A. thaliana*. The normalized transcript levels are the result of two independent biological replicates (*N* = 2). They are presented relatively to the *ArathNictaba* expression level determined in 6-day-old plantlets. Bars represent the mean ± SE normalized relative expression and asterisks indicate statistically significant differences to the expression level of *ArathNictaba* in 6-day-old plantlets (^∗^*p* ≤ 0.05, ^∗∗^*p* ≤ 0.01, ^∗∗∗^*p* ≤ 0.001; REST analysis).

Using RT-qPCR, the expression level of the *ArathNictabas* was investigated in different tissues from *Arabidopsis* during development under standard growth conditions. Plant material was collected at different developmental stages starting from 6-day-old plantlets to 54-day-old plants and the normalized relative expression for the three *ArathNictabas* was quantified throughout the development of the plant relative to the expression of these *ArathNictabas* in 6-day-old plantlets (**Figure 3**). Transcripts for the three *ArathNictaba* genes are detected in every tissue at all developmental stages tested. The expression level of *AN3* is significantly higher in the stems and the flowers compared to the expression in 6-day-old plantlets. The expression level of *AN4* is slightly but significantly higher in the roots and significantly lower in the flowers of the plant compared to its expression in 6-day-old plantlets. The expression level of *AN5* is significantly higher in the rosette- and cauline leaves at all developmental stages tested. Moreover, the expression level of *AN5* is slightly but significantly lower in the flowers compared to its expression in 6-day-old plantlets.

The normalized expression of *AN3*, *AN4*, and *AN5* compared to the expression of one of the three reference genes (*PP2A*, *TIP41*, or *UBC9*) is much lower for *AN3* and *AN4* than for *AN5*, indicating that the expression level of *AN5* is higher than the expression levels of *AN3* and *AN4* in all tissues throughout the development of the plant (data not shown).

### *ArathNictaba* Expression Is Stress-Inducible

#### *AN3, AN4*, and *AN5* Are Differentially Expressed in Response to Hormone Treatments

**FIGURE 4:**
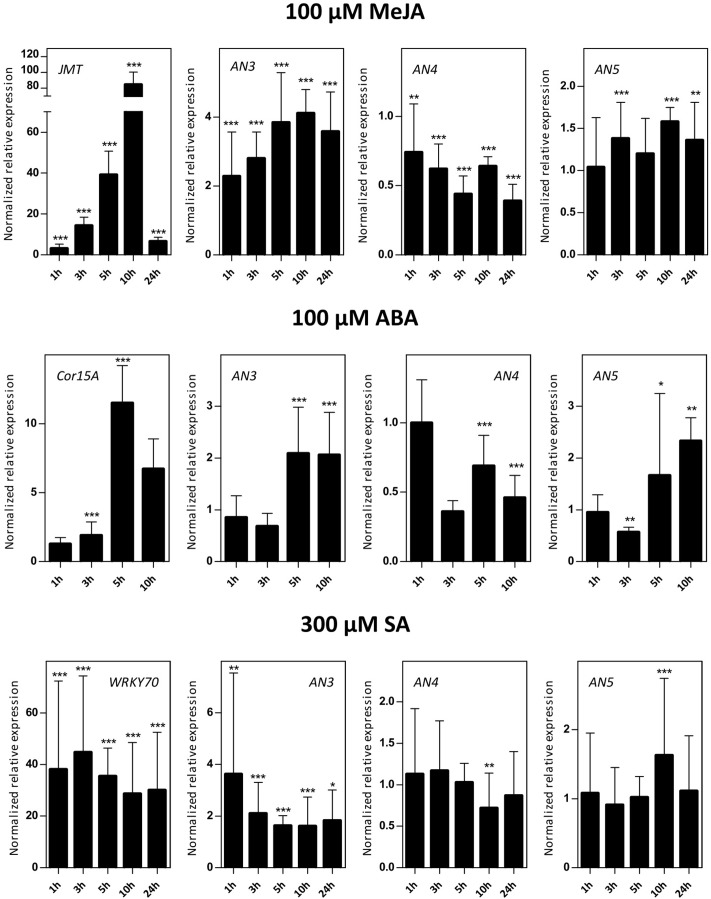
Normalized relative expression of *ArathNictaba* genes after MeJA, ABA, and SA hormone treatment. The normalized transcript levels are the result of two or four independent biological replicates (*N* = 2 for ABA, *N* = 4 for MeJA and SA). They are presented relatively to the *ArathNictaba* expression level determined in the mock treated plantlets. Bars represent the mean ± SE normalized relative expression and asterisks indicate statistically significant differences to the expression level of *ArathNictaba* in mock treated plantlets (^∗^*p* ≤ 0.05, ^∗∗^*p* ≤ 0.01, ^∗∗∗^*p* ≤ 0.001; REST analysis). The normalized relative expression levels for the control genes for each stress are presented in the left panels.

Sixteen-day-old *Arabidopsis* seedlings were subjected to different hormone treatments in particular 100 μM MeJA, 100 μM ABA, and 300 μM SA. Transcript levels for *AN3*, *AN4* and *AN5* were determined by qPCR analysis (**Figure 4**). Control genes known to be responsive to MeJA (*JMT*), ABA (*Cor15A*), and SA (*WRKY70*) treatments are significantly upregulated indicating that the plants sensed the different stress treatments.

The expression of *AN3* is significantly upregulated after treatment with different hormones with a fourfold upregulation after 5, 10, and 24 h of MeJA treatment. After ABA and SA treatment, the upregulation of the expression of *AN3* is less pronounced. ABA treatment resulted in a twofold upregulation after 5 and 10 h, SA treatment yielded a 1.6–3.6-fold upregulation after 1, 3, 5, 10, and 24 h.

In contrast with the expression of *AN3*, the expression of *AN4* is mostly downregulated after treatment with different hormones with a 2–2.5-fold downregulation after 5, 10, and 24 h of MeJA treatment. Whereas a twofold upregulation of the expression of *AN3* was observed after 5 and 10 h of ABA treatment, the expression of *AN4* is approximately two times significantly downregulated. After SA treatment, the expression of *AN4* is not changed except for a small significant downregulation after 10 h.

The expression of *AN5* is only weakly influenced by the MeJA treatment. Similar to the expression of *AN3*, there is a twofold significant upregulation of the expression of *AN5* after 5 and 10 h of ABA treatment. After SA treatment, the expression of *AN5* is not changed except for a small significant upregulation after 10 h.

#### The Expression of the *ArathNictabas* Showed Dissimilar Patterns after Abiotic Stress Treatments

**FIGURE 5:**
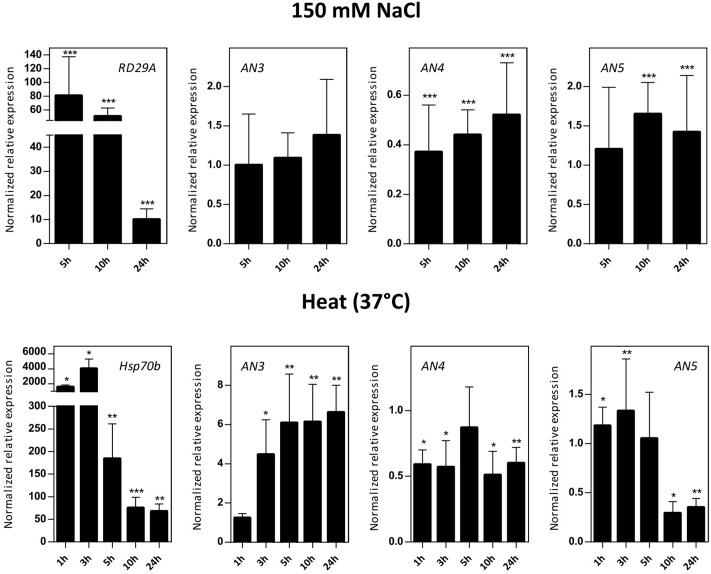
Normalized relative expression for *ArathNictaba* genes after salt and heat stress. The normalized transcript levels are the result of two or four independent biological replicates (*N* = 2 for heat stress, *N* = 4 for salt stress). They are presented relatively to the *ArathNictaba* expression level determined in the mock treated plantlets. Bars represent the mean ± SE normalized relative expression and asterisks indicate statistically significant differences to the expression level of *ArathNictaba* in mock treated plantlets (^∗^*p* ≤ 0.05, ^∗∗^*p* ≤ 0.01, ^∗∗∗^*p* ≤ 0.001; REST analysis). The normalized relative expression levels of the control genes for each stress are presented in the left panels.

Transcript levels for *AN3*, *AN4*, and *AN5* were determined in 16-day-old *Arabidopsis* seedlings subjected to salinity (150 mM NaCl) and heat stress (**Figure 5**). Control genes known to be responsive to salt (*RD29A*) and heat (*Hsp70b*) stress are significantly upregulated indicating that the plants sensed the different abiotic stress treatments. The expression level of *AN3* is not affected by salt stress but is 4–6 times significantly upregulated by heat stress after 3, 5, 10, and 24 h. Overall, the expression of *AN4* is two times downregulated after salt as well as heat stress. The expression of *AN5* is only slightly influenced by salt stress and showed a threefold significant downregulation after 10 and 24 h of heat stress.

#### Expression of the *ArathNictabas* after Different Biotic Stresses

**FIGURE 6:**
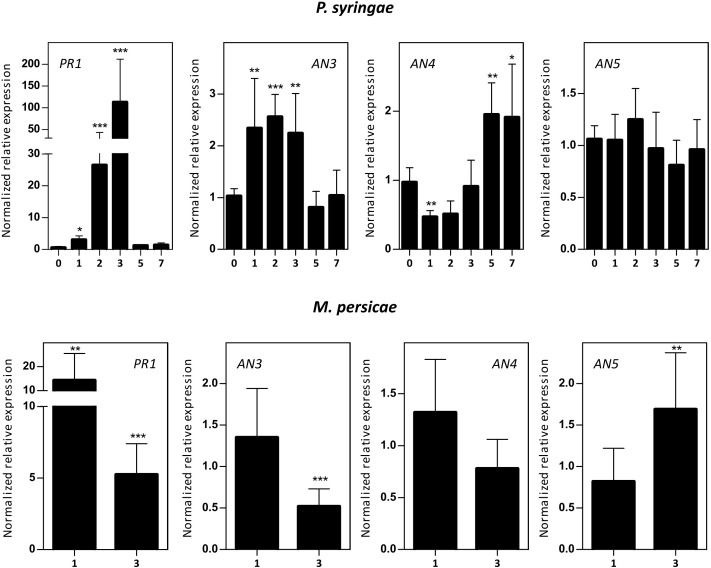
Normalized relative expression for *ArathNictaba* genes after *Pseudomonas syringae* infection and *M. persicae* infestation. The normalized transcript levels are the result of three or four independent biological replicates (*N* = 3 for *P. syringae*, *N* = 4 for *M. persicae*). They are presented relatively to the *ArathNictaba* expression level determined in the mock treated plants. Bars represent the mean ± SE normalized relative expression and asterisks indicate statistically significant differences to the expression level of *ArathNictaba* in mock treated plants (^∗^*p* ≤ 0.05, ^∗∗^*p* ≤ 0.01, ^∗∗∗^*p* ≤ 0.001; REST analysis). Numbers on the *x*-axis represent the number of days after infection/infestation. The normalized relative expression levels of the control genes for each stress are presented in the left panels.

Five-week-old *Arabidopsis* plants were subjected to infection assays with *P. syringae* or *B. cinerea*, and *M. persicae* infestation. Mock/biotic stress treated samples were collected 0–7 days post-infection/infestation (dpi) and transcript levels for *AN3*, *AN4*, and *AN5* were determined (**Figure 6**). qPCR analysis revealed an early 2–2.5-fold upregulation (1–3 dpi) of the expression of *AN3* after *P. syringae* infection and a late twofold upregulation (5 –7 dpi) of the expression of *AN4*. However, the expression of *AN5* did not change after *P. syringae* infection. Fungal infection with *B. cinerea* affected *ArathNictaba* expression levels only weakly (Supplementary Figure 2). A small but significant downregulation of the transcript levels for *AN5* was observed at 2 and 3 dpi. In contrast infestation with *M. persicae* revealed an almost twofold upregulation of the transcript levels for *AN5* after 3 days. The expression of *AN4* is not affected by infestation of the plants with *M. persicae* whereas the expression of *AN3* shows a twofold downregulation after 3 days. Control genes known to be responsive to *P. syringae* (*PR1*), *B. cinerea* (*PDF1.2*), and *M. persicae* (*PR1*) are significantly upregulated, indicating that the plants sensed the different biotic stress treatments.

### *ArathNictaba* Overexpression Lines Show Less Disease Symptoms and Bacterial Growth after *P. syringae* Infection

**FIGURE 7:**
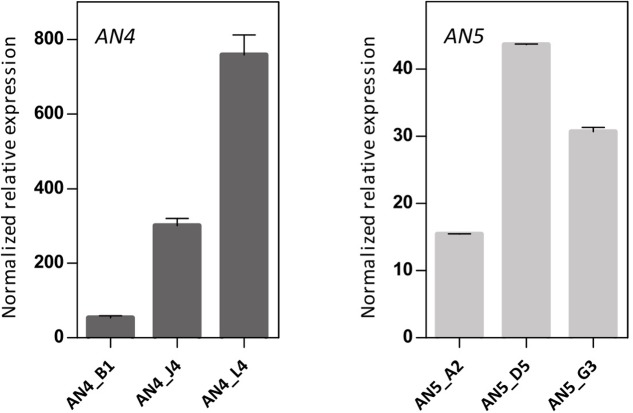
Expression analysis of *ArathNictaba* overexpression level in 2-week-old 35S::*AN4* and 35S::*AN5* transgenic lines. Normalized relative expression of *AN4* and *AN5* compared to WT plants (*N* = 1). Error bars represent standard deviations.

To further investigate the biological importance of the ArathNictabas in the stress response, *A. thaliana* was transformed with the overexpression constructs 35S::*AN4* and 35S::*AN5*, and transgenic lines were selected. Transcript levels for *AN4* and *AN5* were determined in the homozygous *AN4* and *AN5* overexpression lines using RT-qPCR on cDNA obtained from 15-day-old *Arabidopsis* seedlings. The 35S::*AN4* transgenic lines show 50- to 800-fold expression of *AN4* compared to WT plants. However, for the 35S::*AN5* transgenic lines a much lower overexpression level is observed, ranging between 15- and 45-fold compared to WT plants. Based on their expression level relative to the *ArathNictaba* expression in WT plants, three independent overexpression lines exhibiting different overexpression levels were selected (**Figure 7**).

**FIGURE 8:**
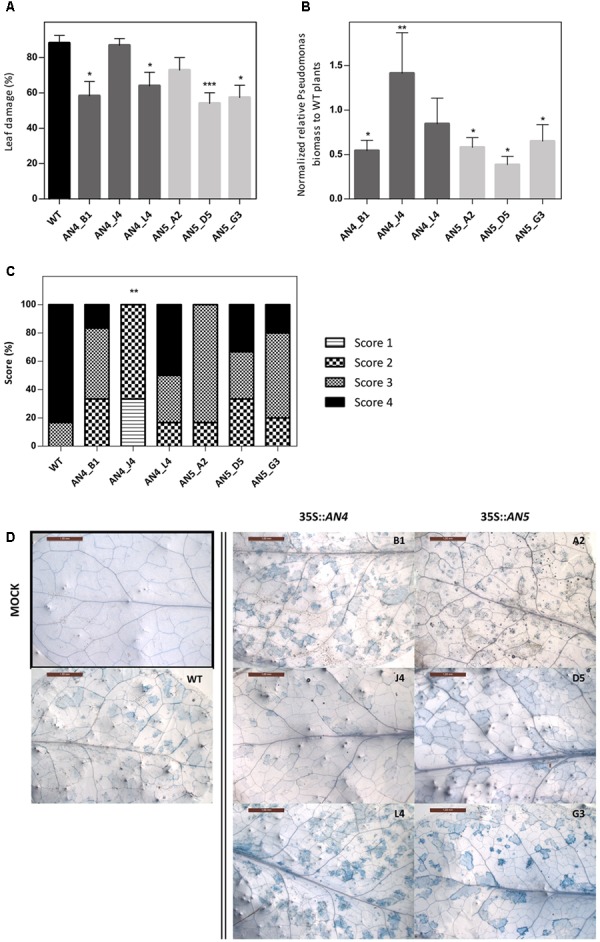
**(A)** Percentage of leaf damage in WT and transgenic overexpression *A. thaliana* plants infected with *P. syringae* at 4 dpi. **(B)** Normalized relative *P. syringae* biomass in the overexpression lines compared to WT plants at 4 dpi. **(C)** Scoring of trypan blue stained leaves **(D)** from overexpression lines compared to WT plants at 4 dpi. **(A)** Percentage of leaf damage calculated with Assess 2.0 at 4 dpi as percent ratio of yellow lesion area relative to the total leaf area. Bars represent the mean ± SE of two independent biological replicates with six individual leaves per line per replicate. Asterisks indicate statistically significant differences to the percentage of leaf damage in WT plants (^∗^*p* ≤ 0.05, ^∗∗^*p* ≤ 0.01, ^∗∗∗^*p* ≤ 0.001; Mann–Whitney *U* test). **(B)** Bars represent the mean ± SE relative *P. syringae* biomass in overexpression lines to biomass in WT plants at 4 dpi from two independent biological replicates normalized with two *A. thaliana* reference genes (*ACT2* and *PEX4*) in REST-384. Asterisks indicate statistically significant differences to the biomass in WT plants (^∗^*p* ≤ 0.05, ^∗∗^*p* ≤ 0.01, ^∗∗∗^*p* ≤ 0.001; pair wise fixed reallocation randomization test REST-384). **(C)** Bars represent the scores in percentage from two independent biological replicates (*N* = 2) with each time three leaves stained per line. Score 1: 0%, score 2: 1–30%, score 3: 31–60% and score 4: 61–100% trypan blue staining or cell death. Asterisks indicate statistically significant differences to the trypan blue staining in WT plants (^∗^*p* ≤ 0.05, ^∗∗^*p* ≤ 0.01, ^∗∗∗^*p* ≤ 0.001; Mann–Whitney *U* test). **(D)** Representative pictures are shown for each transgenic line. Only one picture of a mock treated leaf is shown (black square).

Wild type and transgenic 35S::*AN4* and 35S::*AN5 A. thaliana* plants were infected with the virulent hemibiotrophic *P. syringae* to investigate the role of the *ArathNictaba* genes in the defense against this pathogen. Infection of *A. thaliana* plants with *P. syringae* results in yellow lesion areas on the rosette leaves of the plant. Leaf damage was measured daily on scanned leaves and the percentage of the lesion area relative to the total leaf area was calculated. First bacterial lesions started to appear at 3 dpi, but only at 4 dpi differences in leaf damage were observed for the overexpression lines compared to WT plants (**Figure 8A**). Statistically significant differences in leaf damage compared to the leaf damage in WT plants were observed especially for lines AN4_B1, AN4_L4, AN5_D5, and AN5_G3. These four overexpression lines reveal a significantly lower percentage of leaf damage compared to WT plants suggesting they are more tolerant to *P. syringae* infection. The leaf damage in mock treated plants was also determined but was never higher than 6.5% (data not shown). Furthermore, the leaf damage observed for the 35S::*AN5* lines is significantly (*p* ≤ 0.05) negatively correlated with the expression level of the different overexpression lines (Pearson correlation, SPSS23). No such correlation between the level of leaf damage and the expression level of *AN4* was observed for the different overexpression lines for *AN4*.

To strengthen these results, *P. syringae* biomass on the *A. thalian*a WT and transgenic plants was quantified in leaves at 3 and 4 dpi. Transgenic lines AN4_B1, AN5_D5, and AN5_G3, which showed a significantly lower percentage of leaf damage compared to WT plants, also had a significantly lower *P. syringae* biomass (**Figure 8B**) indicating that these overexpression lines are more tolerant than WT plants to infection with *P. syringae*. Transgenic line AN5_A2, which had less (though not significantly) leaf damage compared to WT, also revealed a significantly lower *P. syringae* biomass. Transgenic line AN4_L4 had a lower (but not significant) *P. syringae* biomass compared to WT plants.

A third analysis included the visualization and quantification of cell death in the leaves using trypan blue staining (**Figures 8C,D**). Transgenic line AN4_B1 showed a significantly lower level of leaf damage and *P. syringae* biomass compared to WT plants (**Figures 8A,B**) which is in agreement with the differences (not significant) observed for cell death (**Figure 8C**) concluding that line AN4_B1 is more tolerant to *P. syringae* infection than WT plants. Surprisingly, line AN4_J4 showed a significantly reduced amount of cell death compared to WT plants (**Figure 8C**) but the percentage of leaf damage was almost equal to that of WT plants and the *P. syringae* biomass was significantly higher than in WT plants (**Figures 8A,B**). Transgenic line AN4_L4 showed comparable results to WT plants in all analyses except for the leaf damage analysis which revealed a significantly lower leaf damage (**Figure 8A**). As such it cannot be concluded that line AN4_J4 and AN4_L4 are more tolerant to *P. syringae* infection. *P. syringae* biomass for line AN5_A2 is significantly lower than for the WT plants (**Figure 8B**) which is in agreement with the lower percentages of leaf damage and cell death, suggesting AN5_A2 is more tolerant to *P. syringae* infection than WT plants. Lines AN5_D5 and AN5_G3 show significantly lower levels of leaf damage and *P. syringae* biomass but the amount of cell death is not significantly lower. Therefore lines AN5_D5 and AN5_G3 are probably also more tolerant to *P. syringae* infection than WT plants. The trypan blue staining in mock treated plants was not quantified since no signs of cell death were observed (**Figure 8D**).

### Pull-Down Analysis to Search for Interacting Partners of AN4

To search for interacting partners, recombinant protein was produced and used for pull-down assays. Recombinant protein production was attempted for AN3, AN4, and AN5 in two eukaryotic systems namely the yeast *Pichia pastoris* and a tobacco cell culture of bright yellow 2 cells. However, no recombinant protein could be detected for either of the ArathNictabas. Recombinant protein production in *E. coli* resulted in a small amount of AN4 protein, but no protein for AN3 and AN5 was detected in the soluble fraction. The pull-down experiments were performed with recombinant AN4 produced in *E. coli* and *Arabidopsis* extracts from WT seedlings or seedlings treated with 150 mM NaCl for 5 h. All *E. coli* and *A. thaliana* proteins were identified using mass spectrometry. Comparative analyses for pull-down assays performed with the IPTG induced *E. coli* culture producing AN4 and a non-induced *E. coli* culture allowed to exclude the *E. coli* proteins that bound aspecifically to the Ni matrix and the *Arabidopsis* proteins that bind aspecifically to these *E. coli* proteins or to the Ni matrix. Results from the pull-down assays with extracts from non-treated and salt treated plants were also compared with each other. Since the transcript levels for AN4 in *Arabidopsis* are downregulated after salt stress (see section “The Expression of the *ArathNictabas* Showed Dissimilar Patterns after Abiotic Stress Treatments”) this can also be the case for the interaction partners for AN4. Possible interaction partners for which the expression is downregulated by salt stress are expected to be absent in the analysis using the plant extract from the salt stressed plants compared with the analysis using the extract from the non-treated *Arabidopsis* seedlings.

**FIGURE 9:**
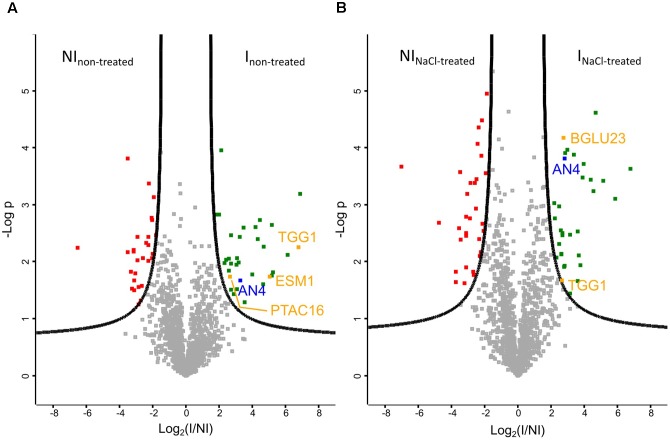
Volcano plots of proteins identified by mass spectrometry (*N* = 3). **(A)** Plant extract from non-treated *Arabidopsis* seedlings. FDR = 0.05. **(B)** Plant extract from *Arabidopsis* seedlings treated with 150 mM NaCl for 5 h. FDR = 0.01. AN4 is indicated in blue, possible interaction partners are shown in orange, *Escherichia coli* proteins which are significantly more present in the pull-down assays performed with the IPTG treated *E. coli* culture (I) in green and *E. coli* proteins which are significantly less present in these pull-down assays are shown in red. NI, non-induced.

Volcano plots allow to quickly identify significant differences between two large datasets by plotting significance (*p*-value) vs. fold change. A first comparative analysis was made for the pull-down assays performed on Ni matrix with the IPTG induced and non-induced *E. coli* culture, using protein extracts of *Arabidopsis* seedlings grown under normal conditions (**Figure 9A**). As expected, AN4 is significantly more present in the pull-down experiments performed with the induced *E. coli* culture. Next to AN4, three *A. thaliana* proteins originating from the plant extract are also significantly more present in particular TGG1, ESM1, and PTAC16, suggesting these proteins are possible interaction partners for AN4. The volcano plot in **Figure 9B** shows a second analysis resulting from the comparison between the proteins identified in the pull-down assays performed with the IPTG induced and non-induced *E. coli* culture, using a plant extract of salt-treated *Arabidopsis* seedlings. AN4 and two *A. thaliana* proteins TGG1 and BGLU23 are significantly more present in the pull-down experiments with the induced *E. coli* culture (**Figure 9B**), suggesting that these two proteins are possible interaction partners for AN4. TGG1 was also retrieved in the first analysis (**Figure 9A**) making it more conclusive to be a true interaction partner for AN4. Compared to TGG1, the data for BGLU23 had a much lower *p*-value (**Figure 9B**). A comparison between the datasets resulting from the pull-down assays performed using the IPTG induced *E. coli* culture and protein extracts from non-treated *Arabidopsis* seedlings and salt-treated *Arabidopsis* seedlings did not yield any significant hits, suggesting that there are no significant differences in AN4-bound proteins from plant extracts of the salt stress treated *Arabidopsis* seedlings compared to WT seedlings (data not shown). All significant hits from the pull-down experiments can be found in Supplementary Tables 6, 7.

## Discussion

### ArathNictabas Are Expressed in the Nucleus and the Cytoplasm

Alignment of the sequences encoding Nictaba from tobacco and the Nictaba domains from the ArathNictaba sequences showed that the tryptophan residues important for carbohydrate binding activity in Nictaba are conserved in all Nictaba domains. However, based on this observation, no conclusions can be drawn with respect to the carbohydrate binding specificity of the ArathNictabas. Stefanowicz et al. (2012) analyzed the carbohydrate specificity of an F-box Nictaba (AT2G02360) protein from *A. thaliana*. The F-box Nictaba sequence contains the tryptophan residues important for sugar binding activity but glycan array analyses yielded results that were very different compared to the GlcNAc binding Nictaba. F-box Nictaba was shown to specifically bind to *N*-acetyllactosamine, Lewis A, Lewis X, Lewis Y and blood type B motifs (Stefanowicz et al., 2012).

**FIGURE 10:**
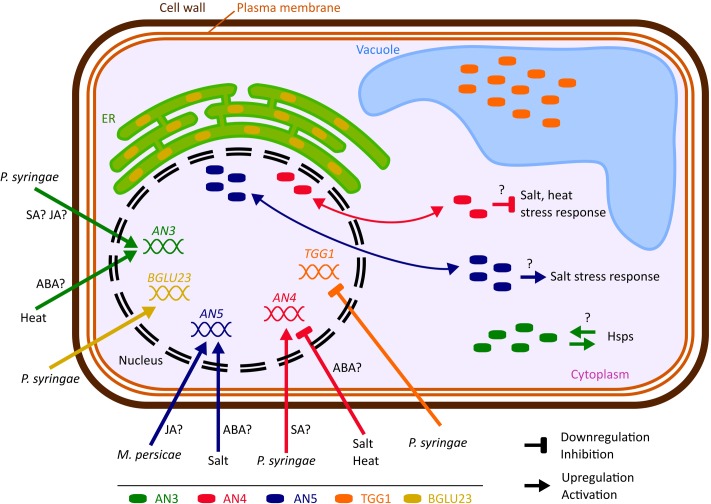
Hypothetical model representing the biological importance of the ArathNictabas in *A. thaliana* cells. The cell and organelles are not drawn to scale. Data available from eFP browser suggest that *BGLU23* is upregulated and *TGG1* is downregulated after *Pseudomonas* infection.

Enhanced green fluorescent protein fusion proteins for AN4 and AN5 localized to the nucleus and the cytoplasm whereas AN3 only resided in the cytoplasm (**Figure 10**). The nucleocytoplasmic localization for AN5 was previously also reported by Cayla et al. (2015). No other experimental data are available with respect to the subcellular localization of AN3 and AN4, but a cytoplasmic localization is also predicted by the SUBA3 server (Hooper et al., 2014).

The results from the microscopy analyses are in agreement with the absence of a signal peptide in the three ArathNictaba sequences and suggest that translation of the ArathNictaba transcripts takes place on free ribosomes in the cytoplasm. AN4 and AN5 locate to the nucleus although the putative NLS reported for the Nictaba sequence from tobacco (K102–K105) is not conserved in the *ArathNictaba* sequences. Lange et al. (2007) reported that proteins larger than 40 kDa are too big to diffuse passively into the nuclear compartment, as already shown in Paine et al. (1975). Görlich (1998) reported that the nuclear pore complex allows passive diffusion from proteins up to 60 kDa. Wang and Brattain (2007) extended this limit, stating that the size of the proteins that diffuse passively into the nucleus can be even larger than 60 kDa. The calculated molecular mass of the EGFP fusion proteins for AN4 and AN5 is 48.5 and 56.3 kDa, respectively. Considering the most recent information about the passive diffusion limit, both proteins could enter the nucleus by passive diffusion through nuclear pore complexes (Wang and Brattain, 2007). Alternatively, AN4 and AN5 possibly contain a non-classical NLS sequence recognized by importin α (Kosugi et al., 2009). Furthermore, additional nuclear import pathways, independent of importin α, have been characterized (Suh and Gumbiner, 2003; Ziemienowicz et al., 2003; Pemberton and Paschal, 2005).

Quantitative analyses of the transcript levels for the *ArathNictabas* in different tissues from *A. thaliana* revealed that the three *ArathNictaba* genes under study are expressed in all tissues throughout development. However, it should be mentioned that lectin expression is generally low, especially for *AN3* and *AN4*. However, specific stress factors can enhance the transcript levels for each lectin. The low expression observed for the *Arathnictabas* is unlike the expression patterns observed for *Nictaba* from tobacco. The tobacco lectin was not detected in plants grown under non-stress conditions (Chen et al., 2002; Lannoo et al., 2007). Similar to *AN3*, *AN4*, and *AN5*, *F-box Nictaba* from *A. thaliana* and the Nictaba-like lectins from soybean were present in plant cells at low levels when grown under optimal growth conditions (no stress), suggesting that the function of the Nictaba homologs is more complex (Stefanowicz et al., 2016; Van Holle et al., 2016).

### The Expression of *ArathNictabas* Is Stress-Inducible

Comparative analyses of the expression patterns for the different Nictaba homologs in response to stress revealed that transcript levels for the *ArathNictabas* are specific and vary depending on the different abiotic or biotic stress treatments performed (**Figure 10**).

#### Abiotic Stress

*Arabidopsis* seedlings were subjected to salt and heat stress, two major abiotic stresses. High salinity stress appears primarily as osmotic stress and as such results in the disruption of homeostasis and ion distribution in the cell (Wang et al., 2003). Heat stress results in the global inhibition of translation and secondly plants have to cope with osmotic and oxidative stress (Wang et al., 2003; Qu et al., 2013; Echevarría-Zomeño et al., 2016). The phytohormone ABA is a key regulator in the plant stress response to osmotic stress (Golldack et al., 2014). Moreover, ABA also plays an important role in heat stress (Vishwakarma et al., 2017).

The expression level of *AN3* is generally highly upregulated after heat stress, up to sixfold, whereas ABA treatment yields a twofold upregulation of the expression level of *AN3* after 5 and 10 h (**Figures 4**, **5**). AN3 possibly plays a role in the heat stress response, dependent or independent of ABA (**Figure 10**). Taking into account that the expression level of *AN3* after salt treatment is not changed, AN3 most probably does not play an essential role in the ABA-dependent salt stress response.

Overall the expression level of *AN4* is downregulated after ABA treatment, salt and heat stress (**Figures 4**, **5**). Downregulation of transcript levels during stress responses is more difficult to interpret, but possibly AN4 has a negative effect on the salt and heat stress response of the plant (**Figure 10**). This negative effect can be due to the repression of specific steps in the stress signaling pathway. Future experiments will have to unravel if AN4 really has a negative impact on the stress responses provoked by salt and heat stress.

The expression level of *AN5* shows a 1.7-fold significant upregulation after salt treatment for 10 h and a 2.3-fold upregulation was observed after treatment with ABA for 10 h (**Figures 4**, **5**). This can be an indication that AN5 plays a role in the ABA-dependent pathway of the salt stress response (**Figure 10**).

#### Biotic Stress

Five-week-old *Arabidopsis* plants were subjected to different biotic stresses (**Figure 6** and Supplementary Figure 2). *P. syringae* is a hemibiotroph that belongs to the Gram-negative plant-pathogenic bacteria (Surico, 2013; Büttner, 2016). *B. cinerea* is a necrotrophic fungus and produces diverse phytotoxic compounds and cell-wall degrading enzymes to induce cell necrosis and as such leakage of nutrients (Mengiste, 2012). *M. persicae* is an insect belonging to the class of pierce-sucking insects and uses its stylet to feed from the phloem of the plant (Louis and Shah, 2013). Two phytohormones are known to play a major role in the defense against biotic stresses. *P. syringae* infection is activating the SA-dependent plant defense pathway while *B. cinerea* infection and *M. persicae* infestation are activating the JA-dependent plant defense pathway (Katagiri et al., 2002; Mengiste, 2012; Pieterse et al., 2012).

The expression level of *AN3* shows an early 2–2.5-fold upregulation after *P. syringae* infection (after 3 days) and a twofold downregulation after 3 days of *M. persicae* infestation. The upregulation upon *P. syringae* infection correlates with the 1.5–3.5-fold upregulation of *AN3* after SA treatment for 1–24 h, but does not correlate with the 2–4-fold upregulation of the expression of *AN3* after 1–24 h MeJA treatment (**Figures 4**, **6**, **10**). For both hormones the expression is significantly upregulated, which is confusing because the SA- and JA-dependent defense pathways normally work antagonistically (Smith et al., 2009; Vos et al., 2013). However, it has to be noted that *P. syringae* produces coronatine, a compound which is structurally similar to JA and was shown to suppress the SA-mediated defense of the plant (Katagiri et al., 2002; Jones and Dangl, 2006). What is more, hormone cross-talk is very complex and also neutral as well as synergistic interactions between SA and JA have been reported. Timing, sequence of initiation and the relative concentration of each hormone play a role in the outcome of the SA–JA cross-talk (Vos et al., 2013).

The expression level of *AN4* is upregulated two times at 5 and 7 days after *P. syringae* infection, but in general the expression level of *AN4* is not changed after SA treatment, indicating that AN4 might play a role in the plant response to *P. syringae* independently of SA (**Figures 4**, **6**, **10**). Yet, only one of the three overexpression lines for *AN4* shows a better tolerance than WT plants toward *P. syringae* infection (**Figure 8**). The better performance of transgenic line AN4_B1 can also be an off-target effect, e.g., related to the site where the T-DNA insertion in the genome took place. The significant downregulation of *AN4* in the MeJA treated *Arabidopsis* seedlings is small and does not correlate with the expression of *AN4* upon *M. persicae* infestation (**Figures 4**, **6**).

The expression level of AN5 shows an almost twofold upregulation after 3 days of *M. persicae* infestation. This is in agreement with the small upregulation of AN5 expression observed after MeJA treatment (**Figures 4**, **6**). Possibly AN5 plays a role in the JA-dependent defense against aphids (**Figure 10**) (Louis and Shah, 2013). This assumption is in agreement with the repression in phloem-feeding activities of *M. persicae* as a result of overexpression of AN5 in *Arabidopsis* (Zhang et al., 2011). Moreover Beneteau et al. (2010) showed that recombinant AN5 at mid-range concentrations, affected weight gain of *M. persicae* nymphs. The SA signaling pathway is also known to be activated during aphid infestation, but this pathway rather facilitates aphid infestation (Louis and Shah, 2013). Transcriptome analysis of *Arabidopsis* subjected to the phloem-feeding insect *Bemisia tabaci*, the silverleaf whitefly, revealed differences in the plant responses after silverleaf whitefly and *M. persicae* infestation. Indeed, also for *AN5* the response is different, since the expression of *AN5* showed a twofold downregulation after *B. tabaci* infestation (Kempema et al., 2006). All overexpression lines for *AN5* are more tolerant (though not always significant) to *P. syringae* infections compared to WT plants. Since all three independent *AN5* overexpression lines show similar results and are more tolerant, this result is probably related to the overexpression of *AN5* (**Figure 8**). At the same time, this result is surprising because the expression level of *AN5* is not changed after *P. syringae* infection.

#### Cross-talk between Abiotic and Biotic Stress

Abiotic and biotic stresses evoke a complex cellular and molecular response in the plant to prevent damage and ensure survival of the plant. Hormones act as signaling molecules in these plant stress responses (Atkinson and Urwin, 2012; Huber and Bauerle, 2016). The phytohormones known to be important in abiotic and biotic stress responses often interact with each other. JA, SA, and ET play a role in abiotic stresses (Miura and Tada, 2014; Dar et al., 2015; Kazan, 2015; Valenzuela et al., 2016), while ABA is also important in the defense against biotic stresses (Tsuda and Katagiri, 2010; Pieterse et al., 2012; Vos et al., 2013). In addition, several growth hormones were also reported to play a role in abiotic and biotic stress, making the hormone signaling in plants subjected to stress even more complex (Tsuda and Katagiri, 2010; Kazan, 2015; Couto and Zipfel, 2016). Moreover, Ca^2+^ and ROS were also shown to play a role in the cross-talk between abiotic and biotic stress (Tsuda and Katagiri, 2010; Bhargava and Sawant, 2013; Couto and Zipfel, 2016; Vishwakarma et al., 2017).

Until now, most studies focused on the effect(s) of a particular stress, but our understanding of the stress signaling pathways under combinations of both abiotic and biotic stresses is still rather poor. In nature however, plants are often simultaneously exposed to multiple stresses. The presence of an abiotic stress can have a positive or a negative impact on the susceptibility to a biotic agent, and vice versa. Moreover, the plant response to multiple stresses can be totally different from the response to each of the individual ones. Therefore, it will be important to study the expression of *ArathNictabas* in plants subjected to a combination of stress factors to develop broad-spectrum stress tolerant crop plants in the future (Atkinson and Urwin, 2012; Rasmussen et al., 2013; Huber and Bauerle, 2016).

### AN4 Interacts with Two Plant Defense-Involved Enzymes

The genome of *A. thaliana* is fully sequenced and annotated. For many proteins, interaction partners are predicted and/or experimentally proven, revealing part of the interactome of *A. thaliana*. Until now, no interaction partners are reported for AN4. Pull-down experiments using AN4-HIS coupled to Ni agarose beads as a bait protein and an *Arabidopsis* extract as prey proteins, followed by mass spectrometry revealed several possible interaction partners for AN4 (**Figure 9**). Two interaction partners were identified in the pull-down assays with the plant lysate originating from the salt stressed *Arabidopsis* seedlings. TGG1 (AT5G26000) or myrosinase 1 is a thioglucoside glucohydrolase localized in the vacuole. BGLU23 (AT3G09260) or PYK10 is a β-*O*-glucosidase localized in the ER bodies. A similar experiment with the plant lysate originating from non-treated *Arabidopsis* seedlings yielded three possible interaction partners, one which was also a significant hit in the first analysis, in particular TGG1. In addition, GDSL esterase/lipase ESM1 (AT3G14210) and protein plastid transcriptionally active 16 (PTAC16; AT3G46780) were identified as possible interaction partners (Supplementary Table 6). ESM1 is a secreted protein with a role in glucosinolate hydrolysis as a myrosinase-associated protein. PTAC16 is known as a chloroplast protein which regulates the membrane-anchoring functions of the nucleoid (Zhang et al., 2006; Burow et al., 2008; Ingelsson and Vener, 2012). It should be mentioned that the latter analysis was the result of two rather than three replicates of the soluble fraction of the non-induced *E. coli* cultures.

Myrosinase 1 belongs to the glycosyl hydrolase 1 family and is a β-thioglucoside glucosidase (Andersson et al., 2009). Glycosyl hydrolases hydrolyse the glycosidic bond between carbohydrates or a carbohydrate and non-carbohydrate moiety (Ahn et al., 2010). The myrosinases from *A. thaliana*, referred to as TGG1 - TGG6 are all grouped in the myrosinase gene family (Barth and Jander, 2006), and catalyze the hydrolysis of glucosinolates, thereby initiating the formation of isothiocyanates, nitriles, thiocyanates, epithionitriles and other reactive products (Thangstad et al., 2004; Andersson et al., 2009). Glucosinolates or thioglucosides consist of a glucose residue linked to an AA derived R-group of aliphatic, aromatic or indole types by a thioglucoside bond (Thangstad et al., 2004). In *A. thaliana*, different glucosinolates with side chains derived from methionine, tryptophan, phenylalanine and isoleucine were found (Barth and Jander, 2006). Myrosinases and glucosinolates are known to be localized in separate plant cells and consequently only make contact after tissue disruption. Myrosinases are present in the myrosin phloem idioblasts in phoem parenchyma while glucosinolates are present in the S-cells adjacent to the phloem (Thangstad et al., 2004; Barth and Jander, 2006; Andersson et al., 2009). After pathogen invasion or insect herbivory, the myrosinases can degrade glucosinolates and as such produce toxic products to protect the plant against these invaders. Thus, myrosinases play a role in plant defense against microbes and herbivores (Barth and Jander, 2006). Next to the myrosin phloem idioblasts, TGG1 was also reported in the guard cells (Thangstad et al., 2004).

BGLU23 or PYK10 is a β-*O*-glucosidase that, like myrosinase 1, belongs to the GH1 family (Nagano et al., 2008). In total 47 β-glucosidases have been identified in *A. thaliana*, called BGLU1 - BGLU47. BGLU23 (or PYK10) and TGG1 (or BGLU38), the two interaction partners identified for AN4, represent two of these β-glucosidases. BGLU23 is a root and hypocotyl specific β-glucosidase which is synthesized with an endoplasmic reticulum (ER) retention signal at its C-terminus (Nitz et al., 2001; Xu et al., 2004). BGLU23 is known to hydrolyze the natural substrate scopolin and other coumarin glucosides similar in structure to scopolin. Scopolin is one of the most abundant secondary metabolites in the *Arabidopsis* roots (Ahn et al., 2010). The resulting scopoletin is a fungitoxic compound and can be polymerized by peroxidase in the presence of H_2_O_2_ (Reigh et al., 1973). This can protect plant cells from the oxidative damage caused by pathogens. BGLU23 localizes to ER bodies in the roots, which are not present in rosette leaves under normal growth conditions. However, ER bodies can be induced by MeJA treatment and wounding in rosette leaves (Nagano et al., 2005; Ahn et al., 2010). Upon disruption of cells, BGLU23 forms large complexes with its binding proteins. One of these binding proteins is PBP1, a jacalin homolog from *A. thaliana* (Ahn et al., 2010). BGLU23 is a glycoprotein with three high-mannose oligosaccharides which can be recognized by PBP1, the jacalin homolog. It is possible that PBP1 participates in the BGLU23 and ER body-mediated defense systems against herbivores and pathogens (Matsushima et al., 2004). PBP1 is thought to act as a molecular chaperone that helps the correct polymerization of BGLU23 when tissues are damaged. This polymerization of BGLU23 is necessary for its activity (Nagano et al., 2005, 2008).

At first sight, there is no obvious link between AN4 and the plant defense-involved enzymes TGG1 and BGLU23. However, interesting observations were made when the localization, the expression pattern and the expression levels were compared in more detail. Protein localization and expression under normal growth conditions can be compared at subcellular level, according to cell type and at tissue level. AN4 resides in the nucleus and the cytoplasm of the cell and its expression under normal growth conditions is significantly higher in the roots compared to 6-day-old seedlings (**Figures 2**, **3**). Jakoby et al. (2008) identified AN4 as one of the 5% most highly expressed genes in mature *Arabidopsis* trichomes. Moreover, they reported a strong enrichment of genes involved in root atrichoblast differentiation in the trichome transcriptome (Jakoby et al., 2008). The sequence encoding TGG1 contains a signal peptide and the protein localizes to the vacuole (Thangstad et al., 2004). Xue et al. (1995) reported the expression of TGG1 in the leaves, stems and floral organs. At cell level, TGG1 was especially found in the guard cells and in phloem myrosin cells. Myrosin cells or idioblasts are specific cells that differ greatly from the neighboring cells in size, structure and content. The morphology of these specific cells can vary in different organs, tissues and developmental stages (Thangstad et al., 2004). BGLU23, like TGG1, is synthesized with a signal peptide, but also contains an ER-retention signal (KDEL sequence). This β-O-glucosidase, also called PYK10 is specifically localized in ER bodies and is root specific (Nitz et al., 2001; Matsushima et al., 2003; Ahn et al., 2010). BGLU23 was also reported in the *Arabidopsis* plasmodesmal proteome, but probably represents a cytoplasmic contaminant in this fraction (Fernandez-Calvino et al., 2011).

A striking similarity between AN4 and BGLU23 at tissue level is their expression in the roots of *Arabidopsis*. However, according to microarray data (eFP browser) the expression level of *BGLU23* in the roots is much higher than the expression level of *AN4* under normal growth conditions (Winter et al., 2007). Furthermore, it is interesting to note that *BGLU23* was also identified as one of the 5% most highly expressed genes in mature *Arabidopsis* trichomes (Jakoby et al., 2008). Although trichomes are present on the leaves, it is reported that trichome development in leaves and atrichoblast development in roots share a network of transcription factors (Pesch and Hülskamp, 2004; Schellmann et al., 2007). However, it is not known whether trichomes and atrichoblasts really share common patterns of gene expression (Jakoby et al., 2008). Jakoby et al. (2008) reported high activity of genes involved in the glucosinolate pathways in trichomes, indicating the role of trichomes in plant defense. Possibly AN4 and BGLU23 can interact in the roots or trichomes when the plant is subjected to stress. To do so, at least one of the two proteins would have to change its subcellular localization. Changes in subcellular localization after stress have been reported before (García et al., 2010; Moore et al., 2011). Alternatively AN4 and BGLU23 can interact as a result of the cell damage provoked by, e.g., pathogens.

Another similarity at tissue level is seen between TGG1 and AN4. TGG1 was especially abundant in the phloem myrosin cells whereas AN4 was reported as a PP2 of *Arabidopsis*. PP2 proteins are most abundant in the phloem sap. Though the sequence of AN4 shows high similarity to the PP2 domain, a protein domain that is conserved among many species in the plant kingdom, there is no evidence for the presence of AN4 in the phloem, except for the microarray data from eFP browser (Dinant et al., 2003; Thangstad et al., 2004; Winter et al., 2007).

The expression level of *AN4* is low under normal growth conditions and its expression remains low or is even downregulated after all investigated stress treatments, except for the *P. syringae* infection. *P. syringae* infection of 5-week-old rosette leaves resulted in approximately two times upregulation of *AN4* transcript levels at the latest timepoints (after 5 and 7 days). According to the microarray data of the eFP browser, *BGLU23* shows a sixfold upregulation 24 h after *P. syringae* infection, but *TGG1* shows a small downregulation (Winter et al., 2007). It has to be mentioned that our data also first show a twofold significant downregulation of AN4 about 24 h after *P. syringae* infection. Although *TGG1* and *AN4* show a similar regulation 24 h after *P. syringae* infection, their absolute expression levels are very different, TGG1 is present in much higher levels in infected *Arabidopsis* leaves than AN4 (**Figure 10**). At present, it remains unclear if the expression of *BGLU23* and *AN4*, and their interaction in the trichomes is part of the defense response against *Pseudomonas*.

Since the pull-down analysis is an *in vitro* analysis and the plant extract contains a mixture of proteins derived from different cell compartments, it is uncertain whether the interaction partners retrieved from the pull-down assay represent true *in vivo* interaction partners for AN4. Additionally, TGG1 and BGLU23 are two proteins that are quite abundant in *Arabidopsis* seedlings, whereas AN4 is expressed at a much lower level. Future experiments are thus necessary to confirm these protein interactions *in vivo*.

To conclude, similar to Nictaba from tobacco the ArathNictabas under study are also localized to the nucleus and/or the cytoplasm (Chen et al., 2002). *ArathNictaba 3–5* are expressed at very low levels in all tissues during the development of *A. thaliana* under normal growth conditions, but their expression is clearly stress-inducible, though specific stresses trigger differential expression of *ArathNictaba 3–5*. Thus, it can be concluded that expression patterns for the *ArathNictabas* are specific and vary for different abiotic or biotic stress treatments (**Figure 10**). Our data suggest that ArathNictabas could play a role in the stress response of *A. thaliana*. Future research is needed to investigate the subcellular localization of the ArathNictabas after plants that have been exposed to stress situations. In addition, analyses with mutant lines can help to decipher the role of the ArathNictabas in the plant stress response. Finally, the interaction of AN4 with TGG1 and BGLU23 has to be confirmed *in vivo* and it has to be shown whether this binding is a protein–protein or a protein–carbohydrate interaction.

## Author Contributions

LE and EVD outlined and designed the study. LE performed the experiments, analyzed and interpreted the data and prepared the manuscript. KS provided some cDNAs of stress experiments and helped with the *P. syringae* stress experiment on WT plants. EVD conceived and supervised the experiments and critically revised the manuscript. All authors have read, revised, and approved the final manuscript.

## Conflict of Interest Statement

The authors declare that the research was conducted in the absence of any commercial or financial relationships that could be construed as a potential conflict of interest. The reviewer CF and handling editor declared their shared affiliation.

## Footnotes

1 http://biotools.umassmed.edu/bioapps/primer3_www.cgi

2 http://www.ebi.ac.uk/Tools/msa/clustalo/

3 http://www.ebi.ac.uk/Tools/psa/emboss_water/

4 https://blast.ncbi.nlm.nih.gov

## Abbreviations

AA: amino acids
ABA: abscisic acid
AN3: ArathNictaba3
AN4: ArathNictaba4
AN4-HIS: C-terminally His6-tagged AN4
AN5: ArathNictaba5
ArathNictabas: Nictaba homologs from *A. thaliana*
CTAB: hexadecyltrimethylammonium bromide
EGFP: enhanced green fluorescent protein
GlcNAc: *N*-acetyl-D-glucosamine
IPTG: isopropyl β-D-1-thiogalactopyranoside
IZ: imidazole
MeJA: methyl jasmonate
MS: Murashige and Skoog
NLS: nuclear localization signal
PAGE: polyacrylamide gel electrophoresis
PB: phosphate buffer
PCR: polymerase chain reaction
PP2A: protein phosphatase 2A
qPCR: quantitative PCR
RT: reverse transcriptase
SA: salicylic acid
SDS: sodium dodecyl sulfate
T-DNA: transferred DNA
TBS: Tris-buffered saline
WT: wild type.
