# Supplementary material for: Nictaba Homologs from Arabidopsis thaliana Are Involved in Plant Stress Responses
Source: Front Plant Sci. 2018 Jan 10;8:2218. doi: 10.3389/fpls.2017.02218 (PMC5767604; doi:10.3389/fpls.2017.02218)
Supplement: Supplementary file 1 [file Data_Sheet_1.DOCX]

Supplementary Material

Nictaba homologs from *Arabidopsis thaliana* are involved in plant stress responses

Lore Eggermont, Karolina Stefanowicz, Els J.M. Van Damme^*^

***Correspondence:** Prof. Dr. Els J.M. Van Damme: [elsjm.vandamme@ugent.be](mailto:elsjm.vandamme@ugent.be)

## Supplementary Figures


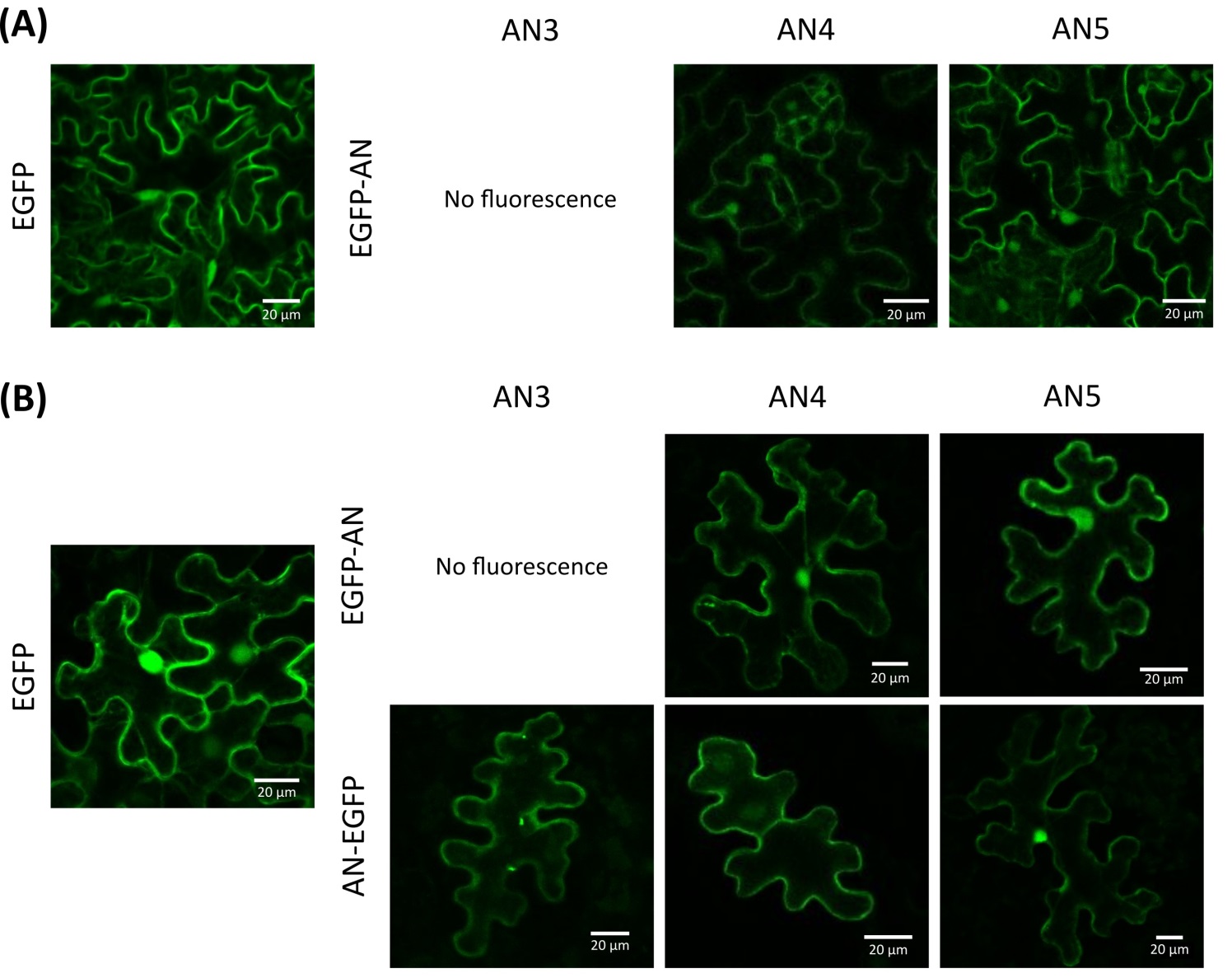


**Supplementary Figure 1.** **(A) Localization of free EGFP and N-terminal EGFP fusion constructs of ArathNictabas expressed in stably transformed *A. thaliana* plants. (B) Localization of free EGFP and N- and C-terminal EGFP fusion constructs of ArathNictabas expressed in transiently transformed *N. benthamiana* leaves.**


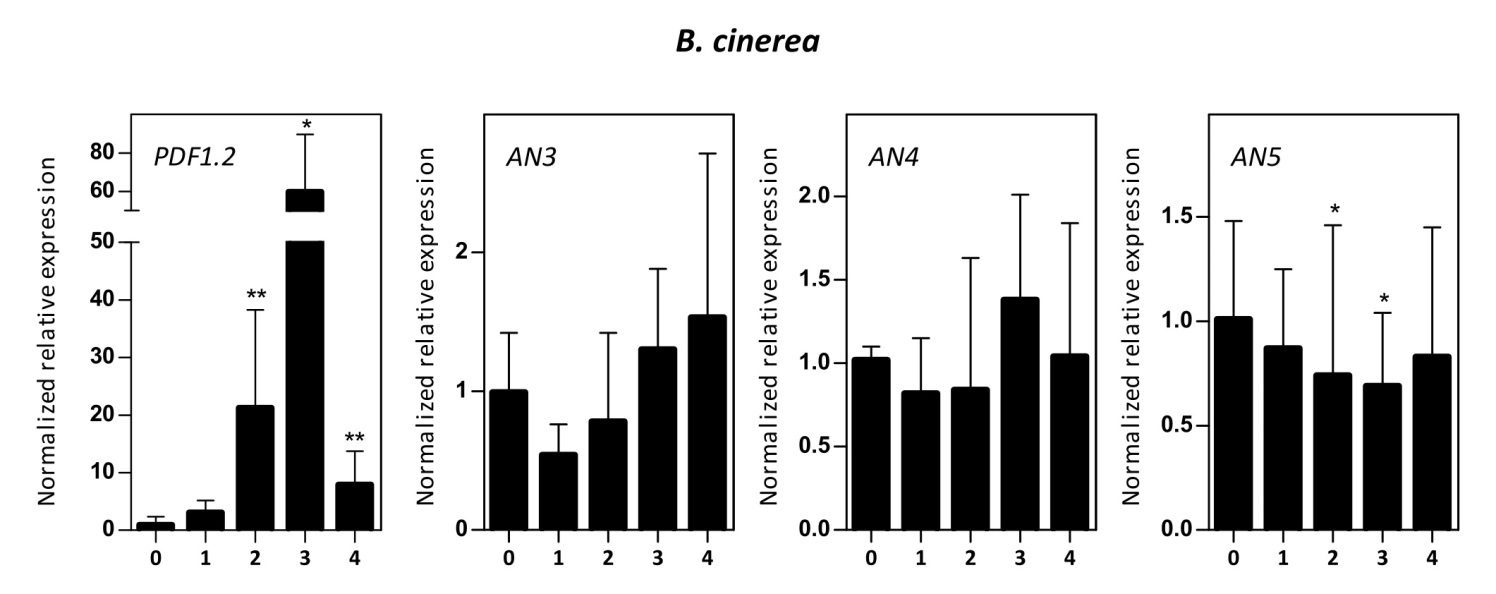


**Supplementary Figure 2. Normalized relative expression for the three *ArathNictaba* genes after *B. cinerea* infection.** The normalized transcript levels are the result of two independent biological replicates (N = 2). They are presented relatively to the *ArathNictaba* expression level determined in the mock treated plants. Bars represent the mean ± SE normalized relative expression and asterisks indicate statistically significant differences to the expression level of *ArathNictaba* in mock treated plants (* p≤0.05, ** p≤0.01, ***p≤0.001; REST analysis). Numbers on the x axis represent the number of days after infection. The normalized relative expression levels of the positive control gene are presented in the left panel.

## Supplementary Tables

**Supplementary table 1 Gene specific primers for cloning.**

| **Target gene** | **Forward primer (5'-3')** | **Reverse primer (5'-3')** |
| --- | --- | --- |
| *AN3* (AT4G19850.2) | evd721 | evd722 |
|  | atgggaataatatggtctatc | tcatgcctcgtgtacataaatc |
| *AN4* (AT1G31200) | evd723 | evd724 |
|  | atgtcttcacaaaagagttcgc | ttacacttcttgaacaaaggc |
| *AN5* (AT4G19840) | evd725 | evd726 |
|  | atgagcaagaaacattgctcag | ttactgtttgggacgaattgc |

**Supplementary table 2 qPCR primers.**

| **Target gene** | **Info** | **Forward primer (5'-3')** | **Reverse primer (5'-3')** |
| --- | --- | --- | --- |
| *AN3* (AT4G19850.2) | / | evd844 | evd845 |
|  |  | TCTTCTCAAAGACAAAGCCACA | GCTTCAAGGAAAAGTCATCGTC |
| *AN4* (AT1G31200) | / | evd846 | evd847 |
|  |  | TTCACAAAAGAGTTCGCATCA | GAGTCACCTCCCCAAACAAA |
| *AN5* (AT4G19840) | / | evd848 | evd849 |
|  |  | GCCACCGGTGACAACTTTAC | GAGGAGAGAGAGATCGGAGGA |
| *PP2A* (AT1G13320) | Reference gene for data normalization | evd727 | evd728 |
|  |  | TCCGAGATCACATGTTCCAAACTC | CCGTATCATGTTCTCCACAACCG |
| *TIP41* (AT4G34270) | Reference gene for data normalization | evd729 | evd730 |
|  |  | TGAACTGGCTGACAATGGAGTG | CATGAGCTTGGCATGACTCTCAC |
| *UBC9* (AT4G27960) | Reference gene for data normalization | evd731 | evd732 |
|  |  | TCCTACTTCATGTAGCGCAGGAC | TCCTCCAGAATAAGGGCTATCCG |
| *JMT* (AT1G19640) | Positive control for MeJA treatment | evd745 | evd746 |
|  |  | TATGTAAGCTCGCCACGATACGCT | AACACGATCAACCGGCTCTAACGA |
| *Cor15A* (AT2G42540) | Positive control for ABA treatment | evd781 | evd782 |
|  |  | CAGTGAAACCGCAGATACATTGGG | GGCTTCTTTTCCTTTCTCCTCC |
| *WRKY70* (AT3G56400) | Positive control for SA treatment | evd811 | evd812 |
|  |  | CATGGATTCCGAAGATCACA | CTGGCCACACCAATGACAA |
| *RD29A* (AT5G52310) | Positive control for NaCl treatment | evd749 | evd750 |
|  |  | ATCACTTGGCTCCACTGTTGTTC | ACAAAACACACATAAACATCCAAAGT |
| *Hsp70b* (AT1G16030) | Positive control for heat treatment | evd735 | evd736 |
|  |  | ATGTATCAGGGTGGTGCTGCT | ACCTCTTCGATCTTGGGACCT |
| *PR1* (AT2G14610) | Positive control for *P. syringae* and *M. persicae* stress | evd1019 | evd1020 |
|  |  | GCTACGCAGAACAACTAAGAGG | GCCTTCTCGCTAACCCACAT |
| *PDF1.2* (AT5G44420) | Positive control for *B. cinerea* infection | evd788 | evd789 |
|  |  | AAGTTGTGCGAGAAGCCAAG | CCATGTTTGGCTCCTTCAAG |
| *ACT2* (AT3G18780) | Reference gene for *P. syringae* biomass | P112 | P113 |
|  |  | GATGAGGCAGGTCCAGGAATC | GTTTGTCACACACAAGTGCATC |
| *PEX4* (AT5G25760) | Reference gene for *P. syringae* biomass | P116 | P117 |
|  |  | TGCAACCTCCTCAAGTTCG | CACAGACTGAAGCGTCCAAG |
| *oprf* (PSPTO_2299) | Pseudomonas gene for *P. syringae* biomass | P508 | P509 |
|  |  | AACTGAAAAACACCTTGGGC | CCTGGGTTGTTGAAGTGGTA |

**Supplementary table 3 EGFP fusion construct primers.**

| **Target gene** | **Construct** | **Forward primer (5'-3')^a^** | **Reverse primer (5'-3')^b^** |
| --- | --- | --- | --- |
| *AN3* (AT4G19850.2) | EGFP-AN3 | evd850 | evd851 |
|  |  | aaaaagcaggcttcatgggaataatatggtctatcttc | agaaagctgggtgtcatgcctcgtgtacataaatc |
| *AN3* (AT4G19850.2) | AN3-EGFP | evd852 | evd853 |
|  |  | aaaaagcaggcttcaccatgggaataatatggtctatc | agaaagctgggtgtgcctcgtgtacataaatc |
| *AN4* (AT1G31200) | EGFP-AN4 | evd769 | evd770 |
|  |  | aaaaagcaggcttcatgtcttcacaaaagagttcgc | agaaagctgggtgttacacttcttgaacaaaggcttc |
| *AN4* (AT1G31200) | AN4-EGFP | evd771 | evd772 |
|  |  | aaaaagcaggcttcaccatgtcttcacaaaagagttc | agaaagctgggtgcacttcttgaacaaaggcttcg |
| *AN5* (AT4G19840) | EGFP-AN5 | evd773 | evd774 |
|  |  | aaaaagcaggcttcatgagcaagaaacattgctcag | agaaagctgggtgttactgtttgggacgaattgc |
| *AN5* (AT4G19840) | AN5-EGFP | evd775 | evd776 |
|  |  | aaaaagcaggcttcaccatgagcaagaaacattgctc | agaaagctgggtgctgtttgggacgaattgc |
| attB1-*ArathNictaba*-attB2 | | evd2 | evd4 |
|  |  | GGGGACAAGTTTGTACAAAAAAGCAGGCT | ggggacaagtttgtacaaaaaagcaggct |

^a^ Underlined nucleotides are the first part of the attB1 gateway cloning site.

^b^ Underlined nucleotides are the reverse complementary of the first part of the attB2 gateway cloning site.

**Supplementary table 4 Primers for overexpression lines.**

| **Target (gene)** | **Info** | **Forward primer (5'-3')** | **Reverse primer (5'-3')** |
| --- | --- | --- | --- |
| *ACT2* (AT3G18780) | Check for gDNA quality | evd280 | evd281 |
|  |  | GGCTGGATTTGCTGGAGATGATGC | GTACGACCACTGGCATACAGGGA |
| *NptII* (pO86A1_p160) | Kanamycin resistance gene | evd463 | evd261 |
|  |  | gaacaagatggattgcacgcagg | TCAGAAGAACTCGTCAAGAAGGCG |
| *ArathNictaba* overexpression constructs | 35S promoter until 35S terminator | evd472 | P1 |
|  |  | GAAACCTCCTCGGATTCCAT | aggtcactggattttggttt |

**Supplementary table 5 AN4 protein expression construct primers.**

| **Target** | **Construct** | **Forward primer (5'-3')^a^** | **Reverse primer (5'-3')^a^** |
| --- | --- | --- | --- |
| *AN4*-Gly3-His6 (AT1G31200) | Gibson-AN4-Gly3-His6-Gibson | P241 | P216 |
|  |  | TTAAGAAGGAGATATACGGGatgtcttcacaaaagagttcgc | GCTTTGTTAGCAGCCGGATCtcaAtgatgAtgatgatgatgTCC |
| pET21a(+) vector | | P242 | P243 |
|  |  | GATCCGGCTGCTAACAAAG | CCCGTATATCTCCTTCTTAAAG |
| pET21a(+) vector (sequencing) | | P258 | P259 |
|  |  | TAATACGACTCACTATAGGG | AAAGGGAATAAGGGCGACAC |

^a^ Underlined nucleotides are the Gibson assembly sites.

**Supplementary table 6 Significant (FDR = 0.05)** **MS hits comparing the pull-down assays using induced non-induced soluble *E. coli* fractions (I/NI), and the plant lysate from non-treated Arabidopsis plants (Figure 9A).**

| **Gene name** | **Protein name** | **Protein ID** | **Origin** | **-Log p** | **Log_2_(I/NI)** |
| --- | --- | --- | --- | --- | --- |
| rpsK | 30S ribosomal protein S11 | A0A140N7L9 | *E. coli* | 3,19 | 6,86 |
| TGG1 | Myrosinase 1 | P37702 | *A. thaliana* | 2,25 | 6,78 |
| ibpA | Small Hsp IbpA | A0A140N1Q5 | *E. coli* | 2,12 | 6,11 |
| ECBD_0490 | Acetyl-CoA carboxylase, biotin carboxyl carrier protein | A0A140N752 | *E. coli* | 1,81 | 5,24 |
| hisB | Histidine biosynthesis bifunctional protein HisB | A0A140NAY3 | *E. coli* | 2,64 | 5,17 |
| hslO | 33 kDa chaperonin | A0A140N2J1 | *E. coli* | 1,75 | 5,16 |
| ESM1 | GDSL esterase/lipase ESM1 | Q9LJG3 | *A. thaliana* | 1,73 | 5,03 |
| ibpB | Small Hsp IbpB | A0A140N3G6 | *E. coli* | 2,27 | 4,69 |
| ileS | Isoleucine-tRNA ligase | A0A140ND98 | *E. coli* | 1,61 | 4,64 |
| ECBD_2906 | DNA-directed RNA polymerase | A0A140NCE7 | *E. coli* | 2,73 | 4,47 |
| hslV | ATP-dependent protease subunit HslV | A0A140NHQ8 | *E. coli* | 2,40 | 4,32 |
| lacZ | β-galactosidase | A0A140NDI2 | *E. coli* | 2,61 | 4,17 |
| dnaJ | Chaperone protein DnaJ | A0A140NFZ9 | *E. coli* | 1,78 | 3,99 |
| rraB | Regulator of ribonuclease activity B | A0A140NDQ0 | *E. coli* | 1,28 | 3,53 |
| valS | Valine-tRNA ligase | A0A140NGV6 | *E. coli* | 2,60 | 3,47 |
| PP2-A9 | Protein PP2-LIKE A9 | Q9SA16 | Recombinant protein | 1,67 | 3,28 |
| ECBD_0346 | Hsp15 | A0A140N4B4 | *E. coli* | 2,44 | 3,23 |
| gpmA | 2,3-bisphosphoglycerate-dependent phosphoglycerate mutase | A0A140N9D9 | *E. coli* | 1,98 | 3,17 |
| yqgE | UPF0301 protein YqgE | A0A140N8C3 | *E. coli* | 1,52 | 3,08 |
| hisD | Histidinol dehydrogenase | A0A140N5W6 | *E. coli* | 2,06 | 3,08 |
| Ppa | Inorganic pyrophosphatase | A0A140NEF6 | *E. coli* | 1,95 | 3,04 |
| ybaB | Nucleoid-associated protein YbaB | A0A140NF80 | *E. coli* | 1,43 | 2,87 |
| **Gene name** | **Protein name** | **Protein ID** | ***Origin*** | **-Log p** | **Log_2_(I/NI)** |
| topA | DNA topoisomerase 1 | A0A140NCX5 | *E. coli* | 1,97 | 2,72 |
| ECBD_1336 | β-ketoacyl synthase | A0A140N9G9 | *E. coli* | 2,46 | 2,71 |
| lexA | LexA repressor | A0A140NHF7 | *E. coli* | 1,51 | 2,70 |
| PTAC16 | Protein plastid transcriptionally active 16, chloroplastic | Q9STF2 | *A. thaliana* | 1,74 | 2,66 |
| hisC | Histidinol-phosphate aminotransferase | A0A140N8D8 | *E. coli* | 2,05 | 2,58 |
| ygiQ | UPF0313 protein YgiQ | A0A140N6D4 | *E. coli* | 1,84 | 2,57 |
| nsrR | HTH-type transcriptional repressor NsrR | A0A140NF33 | *E. coli* | 2,03 | 2,42 |
| mutM | Formamidopyrimidine-DNA glycosylase | A0A140N6H0 | *E. coli* | 1,98 | 2,32 |
| ECBD_2666 | Porin Gram-negative type | A0A140NAN5 | *E. coli* | 3,95 | 2,12 |
| ECBD_2991 | PhoH family protein | A0A140NAC4 | *E. coli* | 2,83 | 2,00 |
| ECBD_0243 | Oligopeptidase A | A0A140N6F6 | *E. coli* | 2,83 | 1,80 |
| ECBD_1563 | Putative PTS IIA-like nitrogen-regulatory protein PtsN | A0A140N8Q5 | *E. coli* | 2,46 | -1,79 |
| ECBD_2279 | Amidohydrolase | A0A140N7L1 | *E. coli* | 3,13 | -1,93 |
| ECBD_1922 | Phosphofructokinase | A0A140NB59 | *E. coli* | 2,14 | -1,98 |
| ECBD_0661 | NADH:flavin oxidoreductase/NADH oxidase | A0A140N7Z9 | *E. coli* | 2,06 | -2,02 |
| ECBD_2590 | NAD(P)H dehydrogenase (quinone) | A0A140N993 | *E. coli* | 2,73 | -2,04 |
| ECBD_2317 | Aldehyde Dehydrogenase | A0A140NAG2 | *E. coli* | 2,77 | -2,08 |
| ECBD_2784 | β-lactamase | A0A140NE46 | *E. coli* | 3,37 | -2,22 |
| ECBD_3373 | Aldehyde oxidase and xanthine dehydrogenase molybdopterin binding | A0A140NDP2 | *E. coli* | 2,02 | -2,26 |
| rssB | Regulator of RpoS | A0A140NAZ4 | *E. coli* | 2,43 | -2,26 |
| ECBD_0641 | Uncharacterized protein | A0A140N7Y1 | *E. coli* | 2,29 | -2,27 |
| ECBD_1055 | Transcriptional regulator, GntR family | A0A140N8Q4 | *E. coli* | 2,33 | -2,38 |
| **Gene name** | **Protein name** | **Protein ID** | ***Origin*** | **-Log p** | **Log_2_(I/NI)** |
| aceK | Isocitrate dehydrogenase kinase/phosphatase | A0A140NHI6 | *E. coli* | 2,06 | -2,60 |
| ECBD_1744 | Trehalose-6-phosphate synthase | A0A140NB73 | *E. coli* | 1,58 | -2,64 |
| ECBD_0868 | PAS modulated sigma54 specific transcriptional regulator, Fis family | A0A140N695 | *E. coli* | 1,32 | -2,72 |
| ECBD_0229 | Transcriptional regulator, LuxR family | A0A140N6Z5 | *E. coli* | 1,55 | -2,85 |
| ECBD_1096 | GCN5-related N-acetyltransferase | A0A140N551 | *E. coli* | 1,25 | -2,86 |
| ECBD_1054 | Peptidoglycan-binding LysM | A0A140N935 | *E. coli* | 2,02 | -2,90 |
| ECBD_1920 | Fructosamine kinase | A0A140N6M2 | *E. coli* | 2,43 | -2,91 |
| ECBD_2922 | Protein TolR | A0A140N9F2 | *E. coli* | 1,79 | -3,03 |
| ECBD_1532 | Transcriptional regulator, MerR family | A0A140N6B8 | *E. coli* | 1,50 | -3,11 |
| ECBD_3372 | Molybdopterin dehydrogenase FAD-binding | A0A140NBF3 | *E. coli* | 1,67 | -3,11 |
| ECBD_0227 | Efflux transporter, RND family, MFP subunit | A0A140N515 | *E. coli* | 2,21 | -3,13 |
| ECBD_3148 | 2-hydroxy-3-oxopropionate reductase | A0A140NC12 | *E. coli* | 2,18 | -3,14 |
| ECBD_1047 | Uncharacterized protein | A0A140N7A6 | *E. coli* | 1,53 | -3,25 |
| ECBD_0225 | Transcriptional regulator, AraC family | A0A140N6E1 | *E. coli* | 1,82 | -3,30 |
| ECBD_2574 | Protein PhoH | A0A140NAZ5 | *E. coli* | 3,81 | -3,49 |
| ECBD_3223 | BolA family protein | A0A140NDA3 | *E. coli* | 2,16 | -3,50 |
| ECBD_3882 | Entericidin EcnAB | A0A140NGM2 | *E. coli* | 2,24 | -6,52 |

**Supplementary table 7 Significant (FDR = 0.01) MS hits comparing the pull-down assays using induced and non-induced soluble *E. coli* fractions (I/NI), and the plant lysate from NaCl-treated Arabidopsis plants (Figure 9B).**

| **Gene name** | **Protein name** | **Protein ID** | **Origin** | **-Log p** | **Log_2_(I/NI)** |
| --- | --- | --- | --- | --- | --- |
| IbpA | Small Hsp | A0A140N1Q5 | *E. coli* | 3,63 | 6,78 |
| rpsK | 30S ribosomal protein S11 | A0A140N7L9 | *E. coli* | 3,10 | 5,86 |
| ibpB | Small Hsp IbpB | A0A140N3G6 | *E. coli* | 3,42 | 5,15 |
| ECBD_2906 | DNA-directed RNA polymerase | A0A140NCE7 | *E. coli* | 4,62 | 4,69 |
| ECBD_0490 | Acetyl-CoA carboxylase, biotin carboxyl carrier protein | A0A140N752 | *E. coli* | 3,24 | 4,56 |
| hisB | Histidine biosynthesis bifunctional protein HisB | A0A140NAY3 | *E. coli* | 3,44 | 4,39 |
| hisD | Histidinol dehydrogenase | A0A140N5W6 | *E. coli* | 3,72 | 3,95 |
| lacZ | β-galactosidase | A0A140NDI2 | *E. coli* | 3,48 | 3,91 |
| fabZ | 3-hydroxyacyl-[acyl-carrier-protein] dehydratase FabZ | A0A140NFC7 | *E. coli* | 1,94 | 3,76 |
| hslO | 33 kDa chaperonin | A0A140N2J1 | *E. coli* | 2,11 | 3,67 |
| hisC | Histidinol-phosphate aminotransferase | A0A140N8D8 | *E. coli* | 2,53 | 3,61 |
| ileS | Isoleucine-tRNA ligase | A0A140ND98 | *E. coli* | 1,66 | 3,60 |
| hslV | ATP-dependent protease subunit HslV | A0A140NHQ8 | *E. coli* | 3,87 | 3,39 |
| ECBD_0180 | Cold-shock DNA-binding domain protein | A0A140N4F2 | *E. coli* | 1,44 | 3,15 |
| ECBD_3970 | Transcriptional regulator, AraC family | A0A140NHD9 | *E. coli* | 2,47 | 3,15 |
| dnaJ | Chaperone protein DnaJ | A0A140NFZ9 | *E. coli* | 3,97 | 2,99 |
| ygiQ | UPF0313 protein YgiQ | A0A140N6D4 | *E. coli* | 3,91 | 2,85 |
| PP2-A9 | Protein PP2-LIKE A9 | Q9SA16 | Recombinant protein | 3,81 | 2,82 |
| ECBD_1042 | Glycine betaine/L-proline ABC transporter, ATPase subunit | A0A140N655 | *E. coli* | 1,93 | 2,82 |
| valS | Valine-tRNA ligase | A0A140NGV6 | *E. coli* | 1,91 | 2,77 |
| BGLU23 | β-glucosidase 23 | Q9SR37 | *A. thaliana* | 4,17 | 2,76 |
| **Gene name** | **Protein name** | **Protein ID** | **Origin** | **-Log p** | **Log_2_(I/NI)** |
| topA | DNA topoisomerase 1 | A0A140NCX5 | *E. coli* | 2,51 | 2,70 |
| rfaH | Transcription antitermination protein RfaH | A0A140NI00 | *E. coli* | 2,46 | 2,67 |
| TGG1 | Myrosinase 1 | P37702 | *A. thaliana* | 1,67 | 2,64 |
| ECBD_1336 | β-ketoacyl synthase | A0A140N9G9 | *E. coli* | 2,14 | 2,63 |
| yqgE | UPF0301 protein YqgE | A0A140N8C3 | *E. coli* | 2,13 | 2,60 |
| ECBD_4231 | Uncharacterized protein | A0A140NDV6 | *E. coli* | 2,54 | 2,60 |
| ECBD_1155 | Iron-sulfur cluster assembly scaffold protein IscU | A0A140N923 | *E. coli* | 2,97 | 2,50 |
| tolB | Protein TolB | A0A140NEI9 | *E. coli* | 2,31 | 2,50 |
| hisH | Imidazole glycerol phosphate synthase subunit HisH | A0A140N8L8 | *E. coli* | 2,07 | 2,33 |
| mutM | Formamidopyrimidine-DNA glycosylase | A0A140N6H0 | *E. coli* | 2,76 | 2,22 |
| ybaB | Nucleoid-associated protein YbaB | A0A140NF80 | *E. coli* | 3,02 | 2,20 |
| ECBD_2317 | Aldehyde Dehydrogenase | A0A140NAG2 | *E. coli* | 4,95 | -1,88 |
| ECBD_2279 | Amidohydrolase | A0A140N7L1 | *E. coli* | 3,56 | -1,92 |
| ECBD_1392 | Uncharacterized protein | A0A140NAA6 | *E. coli* | 2,54 | -2,05 |
| ECBD_0225 | Transcriptional regulator, AraC family | A0A140N6E1 | *E. coli* | 2,66 | -2,12 |
| ECBD_2590 | NAD(P)H dehydrogenase (quinone) | A0A140N993 | *E. coli* | 4,48 | -2,15 |
| ECBD_3998 | Maltose operon periplasmic | A0A140NFH4 | *E. coli* | 3,86 | -2,19 |
| nanA | *N*-acetylneuraminate lyase | A0A140N3N8 | *E. coli* | 2,16 | -2,21 |
| ECBD_2540 | Uncharacterized protein | A0A140N946 | *E. coli* | 2,10 | -2,31 |
| ECBD_0129 | PTS system, mannitol-specific IIC subunit | A0A140N201 | *E. coli* | 2,40 | -2,34 |
| ECBD_1920 | Fructosamine kinase | A0A140N6M2 | *E. coli* | 4,35 | -2,37 |
| rssB | Regulator of RpoS | A0A140NAZ4 | *E. coli* | 4,07 | -2,42 |
| aceK | Isocitrate dehydrogenase kinase/phosphatase | A0A140NHI6 | *E. coli* | 3,45 | -2,48 |
| **Gene name** | **Protein name** | **Protein ID** | **Origin** | **-Log p** | **Log_2_(I/NI)** |
| ECBD_1096 | GCN5-related N-acetyltransferase | A0A140N551 | *E. coli* | 2,93 | -2,52 |
| ECBD_3227 | Cytochrome o ubiquinol oxidase, subunit I | A0A140NA88 | *E. coli* | 3,38 | -2,59 |
| ECBD_0868 | PAS modulated sigma54 specific transcriptional regulator, Fis family | A0A140N695 | *E. coli* | 1,77 | -2,66 |
| ECBD_1962 | SufBD protein | A0A140N985 | *E. coli* | 1,83 | -2,68 |
| ECBD_1047 | Uncharacterized protein | A0A140N7A6 | *E. coli* | 3,38 | -2,84 |
| ECBD_2951 | GTP cyclohydrolase 1 type 2 homolog | A0A140NC19 | *E. coli* | 2,76 | -2,88 |
| ECBD_3372 | Molybdopterin dehydrogenase FAD-binding | A0A140NBF3 | *E. coli* | 3,19 | -3,06 |
| ECBD_3893 | Anaerobic C4-dicarboxylate transporter | A0A140SS54 | *E. coli* | 1,90 | -3,10 |
| acpP | Acyl carrier protein | A0A140NCR5 | *E. coli* | 2,50 | -3,10 |
| ECBD_1822 | PTS system, mannose/fructose/sorbose family, IID subunit | A0A140N9E9 | *E. coli* | 2,45 | -3,10 |
| ECBD_3373 | Aldehyde oxidase and xanthine dehydrogenase molybdopterin binding | A0A140NDP2 | *E. coli* | 2,79 | -3,12 |
| ECBD_0227 | Efflux transporter, RND family, MFP subunit | A0A140N515 | *E. coli* | 1,62 | -3,19 |
| ECBD_2171 | Nitrate reductase, β subunit | A0A140N9U4 | *E. coli* | 2,38 | -3,43 |
| ECBD_2574 | Protein PhoH | A0A140NAZ5 | *E. coli* | 3,57 | -3,46 |
| ECBD_0229 | Transcriptional regulator, LuxR family | A0A140N6Z5 | *E. coli* | 2,59 | -3,53 |
| ECBD_3370 | Uncharacterized protein | A0A140NFC3 | *E. coli* | 1,64 | -3,72 |
| ECBD_2922 | Protein TolR | A0A140N9F2 | *E. coli* | 1,83 | -3,74 |
| ECBD_3148 | 2-hydroxy-3-oxopropionate reductase | A0A140NC12 | *E. coli* | 2,68 | -4,74 |
| ECBD_3882 | Entericidin EcnAB | A0A140NGM2 | *E. coli* | 3,67 | -7,00 |
